# Deep learning-based segmentation and density estimation of corneal nerves and dendritic cells from In Vivo confocal microscopy images

**DOI:** 10.1038/s41598-025-34412-6

**Published:** 2026-01-13

**Authors:** Meichen Ji, Yan Song, Jenny Roth, Ava Dashti, Jorge Lazo, Alisa Lincke, António Filipe Teixeira Macedo, Welf Löwe, Neil Lagali

**Affiliations:** 1 Department of Computer Science and Media Technology, Faculty of Technology, Linnaeus University, Växjö, Sweden; 2 Department of Medicine and Optometry, Faculty of Health and Life Sciences, Linnaeus University, Kalmar, Sweden; 3 Department of Biomedical and Clinical Sciences (BKV), Faculty of Medicine, Linköping University, Linköping, Sweden; 4 Center of Physics-Optometry and Vision Science, University of Minho, Braga, Portugal

**Keywords:** Deep learning, Confocal microscopy, Cornea, Nerve fibers, Dendritic cells, Segmentation, dDensity, COVID-19, Computational biology and bioinformatics, Diseases, Medical research

## Abstract

The purpose of this study was to compare manual assessment of corneal nerve fiber length (CNFL) and dendritic cell (DC) density with an automated assessment method utilizing deep learning segmentation to perform rule-based density estimation. Corneal images were acquired using in vivo confocal microscopy (IVCM) from 100 participants with persistent ocular symptoms after mild COVID-19 (Group 1) and 30 controls without symptoms (Group 2). In total, 1,300 IVCM images were selected and manually annotated for CNFL, and 1,300 for DCs (with dendrites and without dendrites), using FIJI tools. The between-method difference in mean CNFL density was 0.2 $\hbox {mm/mm}^2$ (95% CI: [0.09, 0.23]) for Group 1 and −0.2 $\hbox {mm/mm}^2$ (95% CI: [−0.34, −0.10]) for Group 2. For Group 1, the mean difference for DCs with dendrites was −1.1 $\hbox {cells/mm}^2$ (95% CI: [−1.78, −0.39]), and for DCs without dendrites it was −3.1 $\hbox {cells/mm}^2$ (95% CI: [−5.1, −1.0]). For Group 2, the mean difference for DCs with dendrites was −1.0 $\hbox {cells/mm}^2$ (95% CI: [−1.79, −0.27]), and for DCs without dendrites it was 0.3 $\hbox {cells/mm}^2$ (95% CI: [−1.93, 2.60]). Both manual and automated methods showed significant between-group differences for CNFL (*p*=0.012 and *p*=0.034, respectively) and DC densities (*p*=0.005 and *p*=0.010). The automated approach performed comparably to manual assessment, supporting its potential for reliable, scalable analysis of CNFL and DC in IVCM images.

**Supplementary Information:**

The online version contains supplementary material available at 10.1038/s41598-025-34412-6.

## Introduction

Ocular surface symptoms such as dryness, irritation, and blurred vision are commonly reported in both general eye disorders and following systemic viral infections. Notably, many individuals, after recovering from COVID-19 infection, report persistent ocular symptoms, raising concerns about the virus’s potential impact on the corneal neuroimmune interface^1,2^. The corneal subbasal nerve plexus, responsible for maintaining corneal sensitivity and epithelial integrity, may be disrupted in such cases, contributing to the onset of neuropathic-like eye discomfort^3^. At the same time, dendritic cells (DCs), which are resident antigen-presenting immune cells in the cornea, can become activated in response to inflammation or infection. These cells are typically categorized as DC with dendrites or DC without dendrites, where the former generally exhibit longer dendritic extensions and higher immunological activity, often correlating with active immune responses^4^. Importantly, recent intravital imaging studies indicated that many cells previously categorized as DC without dendrites likely represent T-cells rather than true DCs^5^. This emerging insight may account for some of the morphological variability observed in our dataset and highlights the importance of more refined immune-cell classification in future automated approaches. Together, changes in corneal nerve fiber length (CNFL) and DC density may reflect underlying mechanisms driving ocular symptoms in people that recovered from COVID-19 infections. Among the available corneal nerve and dendritic cells parameters, CNFL and DC density are the most widely used and consistently reported, with CNFL appearing in 94% of studies evaluating corneal nerves in diabetes^6^. In this study, we focus on CNFL and DC density as a starting point toward broader automated analysis of both nerve and immune-cell features in IVCM images.

In vivo confocal microscopy (IVCM) enables non-invasive, high-resolution imaging of both the subbasal nerve plexus and dendritic cells, offering a valuable diagnostic tool for identifying neuroimmune alterations on the ocular surface^7,8^. A common standard for assessing CNFL and DC density using IVCM involves manual annotations of nerve fibers and DCs using the NeuronJ and CellCounter plugins in FIJI software. However, this approach is time-consuming and has poor reproducibility. This problem becomes more significant when attempting to identify DCs, particularly when large numbers of such cells appear within a single image. An alternative is to use an automated approach that applies deep learning models for nerve segmentation and DC detection, followed by a rule-based method to estimate CNFL and DC densities. Previous studies showed high accuracy in segmenting nerve fibers (0.86 sensitivity and 0.90 specificity^9^, 0.84 sensitivity^10^, 0.96 sensitivity and 0.75 specificity^11^) and in detecting DCs (0.89 sensitivity and 0.94 specificity^9^, 0.93 sensitivity and 0.81 specificity^12^) using deep learning.

However, previous studies have been limited by small datasets, and only a few studies have attempted to automate the detection of dendritic cells in IVCM images. Moreover, density calculations were typically performed per image rather than per participant, which may not be appropriate for addressing a clinical question.

In this study, we therefore aimed to compare the manual assessment of corneal subbasal nerves and dendritic cells using NeuronJ and CellCounter in FIJI^13^ to an automated assessment combining deep learning segmentation and rule-based density estimation. Since existing automated tools largely focus on nerve parameters alone, we included ACCMetrics^14^ only as a secondary comparator. ACCMetrics provides established metrics for corneal nerve analysis but lacks support for dendritic cell quantification; therefore, manual expert annotation has served as the primary reference standard for evaluating both neural and immune-cell features, with ACCMetrics used only as an additional benchmark for nerve-related outcomes. To this end, we examined images from a sample of subjects who experienced persistent eye symptoms following mild COVID-19 infection and compared them with a control group of subjects who had previously contracted COVID-19 but had no ocular symptoms and were not hospitalized. The proposed automated assessment was implemented as an extension to our existing deep learning-based decision support system (DSS), named IVCMAssist^15^, and is available for medical researchers to automatically assess the density of CNFL and DC on a per-subject basis.

## Materials and method

### Clinical condition and motivation

As part of a larger clinical study examining ocular symptom development after mild COVID-19 infection, a group of 100 subjects aged over 12 years with ocular symptoms lasting more than 12 weeks after the onset of confirmed COVID-19 infection who had no prior ocular symptoms or eye surgery were included in the study (Group 1). Mild COVID-19 is defined as cases that did not require hospitalization, in line with commonly used clinical criteria. The time between infection and imaging varied from a minimum of 3 months (to conform to the definition of long COVID) to a maximum of 40 months. Over half of the participants with persisting eye symptoms were examined 24 months or more following the initial infection. Exclusion criteria included hospitalization for COVID-19 or post-COVID sequelae, prolonged contact lens wear, systemic disease (e.g., diabetes), prior ocular surgery (e.g., cataract) or other ocular pathology (e.g., glaucoma). Additionally, 30 subjects with prior COVID-19 infection but without hospitalization or ocular symptoms prior were included as a control group (Group 2). The control group, although previously infected with COVID-19, served as a practical healthy control group in this study due to challenges in distinguishing truly COVID-negative individuals during the pandemic, given possible asymptomatic cases and vaccine-induced antibodies. As the control group had prior infection, the potentially confounding variable of COVID-19 itself was mitigated, allowing us to focus only on changes arising in relation to eye symptoms and not the infection alone. Both groups provided signed written informed consent for participation in the study, of which IVCM examinations were included to examine possible neurological and inflammatory biomarkers. For participants under the age of 18, both the participant and parent or legal guardian provided written informed consent to participate. The study protocol was approved by the Swedish Ethical Review Authority (Protocol No. 2022-00365-01) prior to study initiation and recruitment. The study followed the tenets of the Declaration of Helsinki.

### Data collection

This study used anonymized IVCM images collected at the Department of Ophthalmology, Institute for Biomedical and Clinical Sciences, Linköping University, between May 2022 and April 2023. Images were acquired by a single experienced operator using a Heidelberg HRT3-RCM system, capturing the corneal epithelium, subbasal nerve plexus, stromal, and endothelial layers. Sequences of 100 images were captured at 5-8 frames per second (fps) and saved in 8-bit .tif format (384$\times$384 pixels, 400$\times$400 µm) in OS (left eye) and OD (right eye) folders. The folders containing all raw images were uploaded to our IVCMAssist system^15^, which automatically filtered out noisy images and classified the remaining ones into five categories: epithelium, stroma, nerve plexus, endothelium, and oblique slices (the last representing orientation artefacts rather than a true corneal layer). For each participant, images from the subbasal nerve plexus layer were downloaded and reviewed. The representative images were then selected and labeled by an experienced observer, an optometrist with several years of prior experience in published IVCM research^15^.

In total, 1,300 images of corneal nerve fibers (CNF) were selected as representative samples - 10 non-overlapped images with high quality per participant from the central subbasal nerve plexus (5 per eye). The observer was aware of the participants’ group assignments; however, selection was based solely on technical image quality and the requirement for non-overlapping images of the central subbasal nerve plexus representing a broad area of the central cornea and not focused on a specific region, thereby minimizing potential bias. For model training, all images selected by the observer were used. Some images from two participants were excluded from the study due to lack of usable/informative images.

In total, 1,300 images of dendritic cells were selected as representative samples - 10 non-overlapped images with high quality per participant (5 per eye) by the same experienced observer. For model training, all images selected by the observer were used. Two participants were excluded due to a lack of usable images, resulting in a final cohort of 99 participants in Group 1 (990 images) and 29 in Group 2 (290 images). Some images were further excluded for two reasons. In most cases, images are excluded due to lost or corrupted annotation files. This makes the original annotation unrecoverable and renders the image useless. In other rare cases, the observer had not annotated all visible DCs, due to overlapping cells or extremely high densities of DC in particular regions. The automated approach’s data preprocessing steps were unable to process these annotations to properly represent the presence of DCs in these images, leading to the exclusion of these images.

### Manual method

**Fig. 1:**
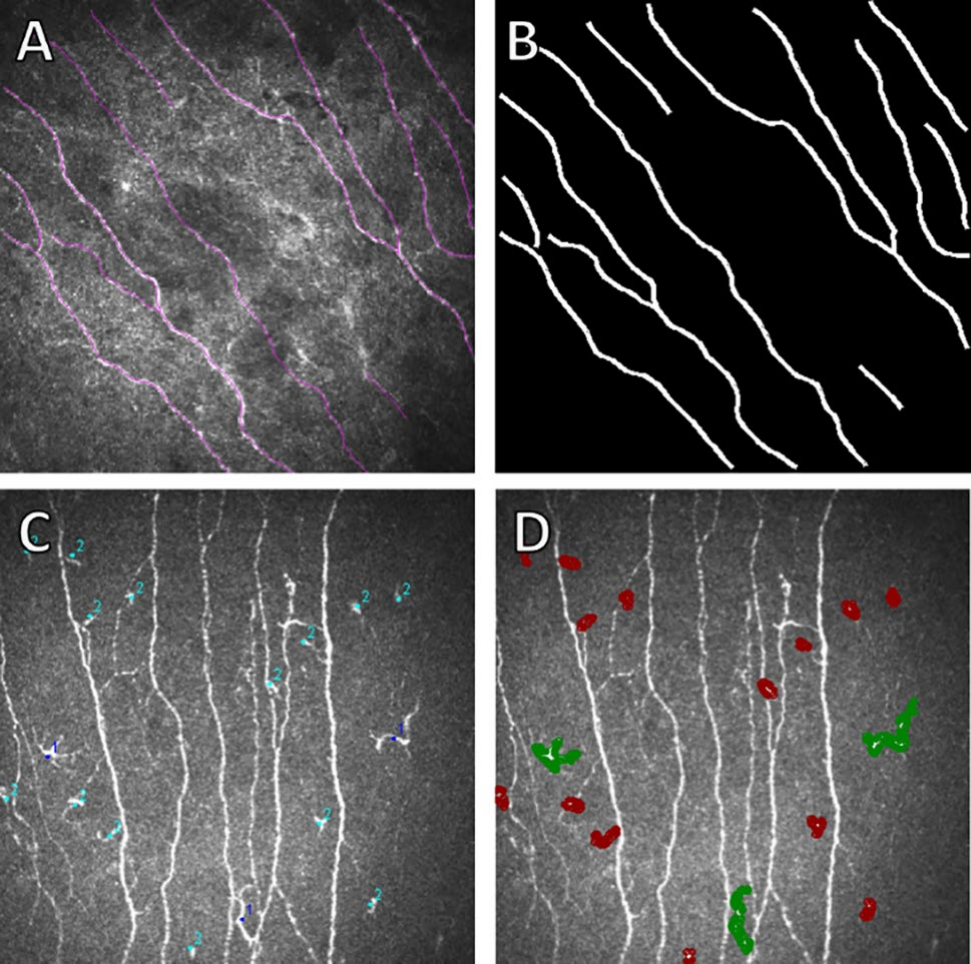
Example of re-annotated images and their original annotations: A - Subbasal nerve fibers annotations using NeuronJ in FIJI; B - Nerve fibers re-annotation where white lines represent the nerves fibers; C - Dendritic cell annotations using CellCounter in FIJI; D - Dendritic cell re-annotation where green regions represent cells with dendrites and red regions represent cells without dendrites.

The selection criteria for images to be annotated for nerve fibers were: well-focused, good contrast and visibility of nerves, vertical and parallel nerves if possible, and representing a single layer of cornea (not oblique with epithelial cells or keratocytes visible). Images for dendritic cell annotation were chosen based on visual assessment, selecting images from different central corneal regions that subjectively best reflected the participant’s typical cell density when considering the entire IVCM examination. Specifically, dendritic cells were annotated when they appeared as highly reflective structures distinct from nerves, with a short, irregular, elongated, or ovoid-shaped cell body, either displaying clear dendrites or lacking dendrites, and located within the subbasal nerve plexus layer. Nerve fiber annotations were produced by manually tracing nerve paths using the NeuronJ plugin in FIJI, and all annotated files were saved in .tif and .ndf formats (see Fig. 1A). These .ndf files were automatically reconstructed in a program to produce masks used for training of the segmentation model (see Fig. 1B). Dendritic cells were annotated using the CellCounter counting tool in FIJI, with label 1 assigned to DCs with dendrites and label 2 to DCs without dendrites (see Fig. 1C). These files were saved as images (.tif) and re-annotated in the Labelme tool^16^, where cells are highlighted with polygons to produce masks for machine learning (see Fig. 1D). It is important to note that annotating dendritic cells in images with high cell density can be challenging, and as illustrated by the structures unintentionally missed in Fig. 1C-D, individual cells may occasionally be overlooked due to human factors, demonstrating both the inevitability of minor annotation errors in large datasets and the value of automated or AI-assisted approaches for large-scale IVCM analysis.

The NeuronJ plugin in FIJI provided the total nerve length in millimeters (mm), values were saved for each image and participant. For each participant, the CNFL density was calculated as follows:

1 $$\begin{aligned} \text {CNFL per participant} = \frac{\text {AVG nerve fiber lengths (in mm)}}{\text {Area of the region analyzed (in mm}^2\text {)}} \end{aligned}$$

where, AVG is an average of 10 images per participant, a single value of CNFL per participant was used for statistical analysis.

CellCounter in FIJI was used to count DCs both with dendrites and without dendrites; values were saved for each image and participant. DC densities were calculated using the following formulas:

2 $$\begin{aligned} \text {Density of DC without dendrites} = \frac{\text {AVG number of DC without dendrites cells counted}}{\text {Area of the region analyzed (in mm}^2\text {)}} \end{aligned}$$

3 $$\begin{aligned} \text {Density of DC with dendrites} = \frac{\text {AVG number of DC with dendrites cells counted}}{\text {Area of the region analyzed (in mm}^2\text {)}} \end{aligned}$$

4 $$\begin{aligned} \text {Total DC Density} = \text {Density of DC without dendrites} + \text {Density of DC with dendrites} \end{aligned}$$

where, AVG is an average of 10 images per participant, a single value of each density per participant was used for statistical analysis. The “area of the region analyzed” was always 0.16 $\hbox {mm}^2$, which corresponds to the area of each image.

### Automated methods

#### Deep learning

The annotated images in the nerve fiber dataset were processed along with their respective .ndf files produced using experienced observer annotations, in which lines were drawn to represent the identified nerve fibers. The .ndf files contain the coordinates of the points that define the line. For the construction of masks for the segmentation model, the lines were reconstructed and drawn with a width of 4 pixels using the ImageDraw.draw method from the Python Imaging Library (PIL)^17^ to resemble the thickness of nerve fibers observed in the IVCM images (see Fig. 1B).

The images in the dendritic cell dataset were re-annotated using the Labelme tool^16^, where polygons were drawn to indicate the location and shape of identified dendritic cells (see Fig. 1D), then saved in .json files containing the bounding coordinates for each annotated polygon. The files were then used to generate masks for the segmentation model. Annotated polygons were drawn onto the masks with their respective class, represented through one-hot encoding. The masks are 3-channel 384*384 images, where one channel represents one class. Pixels with value 1 in the first channel represent the background, value 1 in the second channel represents DCs with dendrites, and value 1 in the third channel represents DCs without dendrites.

The nerve fiber segmentation model, illustrated in Fig. S1 in the Supplementary Material, uses the ResUNet architecture^10^, a hybrid deep learning architecture that integrates residual learning principles with the U-Net framework^18^ widely established in biomedical image segmentation^19^. This architectural choice is well-suited for subbasal nerve fiber segmentation, where capturing both the fine structures of individual nerve fibers and their spatial relationships within the corneal tissue is critical.

The architecture of the dendritic cell segmentation model, illustrated in Fig. S2 in the Supplementary Material, features a similar ResUNet structure previously used to segment subbasal nerve fibers. Detailed information regarding the specifics of these segmentation models can be found in the supplementary methodology.

The models were evaluated using 5-fold cross-validation based on subject stratification, with performance assessed through several metrics. The Dice coefficient^20^ was used to measure the spatial overlap between predicted and ground truth segmentations of subbasal nerve fibers and DCs, where higher values indicate better alignment of predicted regions with actual structures and emphasize true positives. Recall (or sensitivity)^20^ assessed the model’s ability to correctly identify actual positives, reflecting how well subbasal nerve fibers or DCs were segmented when present. Precision^20^ measured the proportion of predicted positive pixels, such as predicted dendritic cells, that were correct, while specificity^20^ quantified the proportion of actual negatives that were correctly identified.

The output of the segmentation models needed to be further processed to estimate the density of CNFL and DCs. To achieve this, two Python algorithms were developed to algorithmically estimate CNFL and DC density. In addition, a machine learning model was implemented as an alternative to the algorithmic CNFL estimation, aiming to compensate for the pixel-wise segmentation errors for CNFL estimation. CNFL is initially estimated in millimeters and then converted to subbasal CNFL density measured in $\hbox {mm/mm}^2$ using formula 1. Since DC density is based on the count of cells rather than a pixel-wise density calculation, there was no need to automate it using machine learning.

#### ACCMetrics

In addition, our automated approach was compared with an existing, well-established nerve fiber image analysis tool, ACCMetrics^14^, which provided the CNFL density for both Group 1 and Group 2. The outputs generated by ACCMetrics were stored in .txt format, and the CNFL densities were extracted for comparison with the manual assessment and our automated approach.

#### Implementation details

The models were implemented using the PyTorch library through Keras^21,22^, in Python 3.10. Model training, evaluation, and deployment were performed on an Ubuntu server 22.04 with a single NVIDIA RTX 4000 Ada Generation GPU, with CUDA version 12.2. The automated pipelines for CNFL and DC were incorporated into the existing IVCMAssist system, which was based on the Angular single-page application framework, with a Python backend using the Flask web-based framework.

### Statistical analysis

Independent samples t-tests were performed for CNFL, with equality of variances assessed using Levene’s Test. As no significant differences in variance were detected (p > 0.05), t-tests assuming equal variances were applied. For the DC subtype analyses, p-values were obtained from the Type III F-test of fixed effects in a linear mixed model with Satterthwaite degrees of freedom, and subsequently adjusted for multiple comparisons using the Benjamini-Hochberg procedure. Effect sizes were calculated for all statistical comparisons. For the independent samples t-test of CNFL, Cohen’s d was computed using pooled standard deviations. For the linear mixed model analyses of DC densities, standardized effect sizes (Cohen’s d) were calculated using the estimated marginal mean differences divided by the residual standard deviation. These statistical analyses were conducted to demonstrate that the automated approach produces statistically meaningful measurements; however, they were not intended to test a specific clinical hypothesis, which would require more balanced subject groups and a different study design. Excel sheets containing the results of manual annotation of CNFL and DCs were imported into SPSS^23^ for the statistical analysis of densities, while in the automated methods, densities were saved in .csv (.txt for ACCMetrics) format and analyzed using Python 3 with the SciPy library.

## Results

After applying image selection and exclusion criteria, 1,280 CNFL images and 1,257 DC images were included in the final analysis.

### Selection of CNFL estimation method

Two methods of estimating CNFL from segmentation model outputs were implemented, and only the best method was integrated into the pipeline. To evaluate the accuracy of algorithmic CNFL estimation from segmented nerve fibers against the machine learning approach (LERA-Net), we computed the mean absolute percentage error (MAPE) and mean absolute error (MAE) across the entire dataset for both approaches. More information, including implementation details and performance metrics, are available in Supplementary Materials.

Shown in Figure S17A, the algorithmic method tended to slightly overestimate Group 1, while Group 2 was slightly underestimated. These errors are mainly caused by the algorithm’s sensitivity to the segmentation model’s errors. This method has a MAPE of 4.04% and an MAE of 0.11. By contrast, the machine learning method, illustrated in Figure S17B, reduced the overestimation of CNFL in Group 1, leading to lower error variance in this group. It also yielded lower overall error, while demonstrating better generalization and robustness. Evident by the lower MAPE and MAE values of 3.43% and 0.09. Therefore, the machine learning approach (LERA-Net) was chosen for integration into the automated CNFL estimation pipeline.

### Manual vs. Automated method

#### CNFL density estimation

As shown in Table 1, the image-wise subbasal nerve fiber segmentation model achieved good performance in recall, precision, and specificity, with a high Dice coefficient indicating a good overlap with the ground truth in segmenting nerve fibers.

**Table 1:** Mean performance (± SD) results across 5-fold cross-validation of the nerve fibers and dendritic cells segmentation models.

	Dice coefficient	Recall (Sensitivity)	Precision	Specificity
Nerve Fibers Segmentation Model	0.904 ± 0.002	0.815 ± 0.011	0.833 ± 0.007	0.987 ± 0.001
DC Segmentation Model	0.635 ± 0.009	0.495 ± 0.020	0.602 ± 0.031	0.999 ± <0.001

**Fig. 2:**
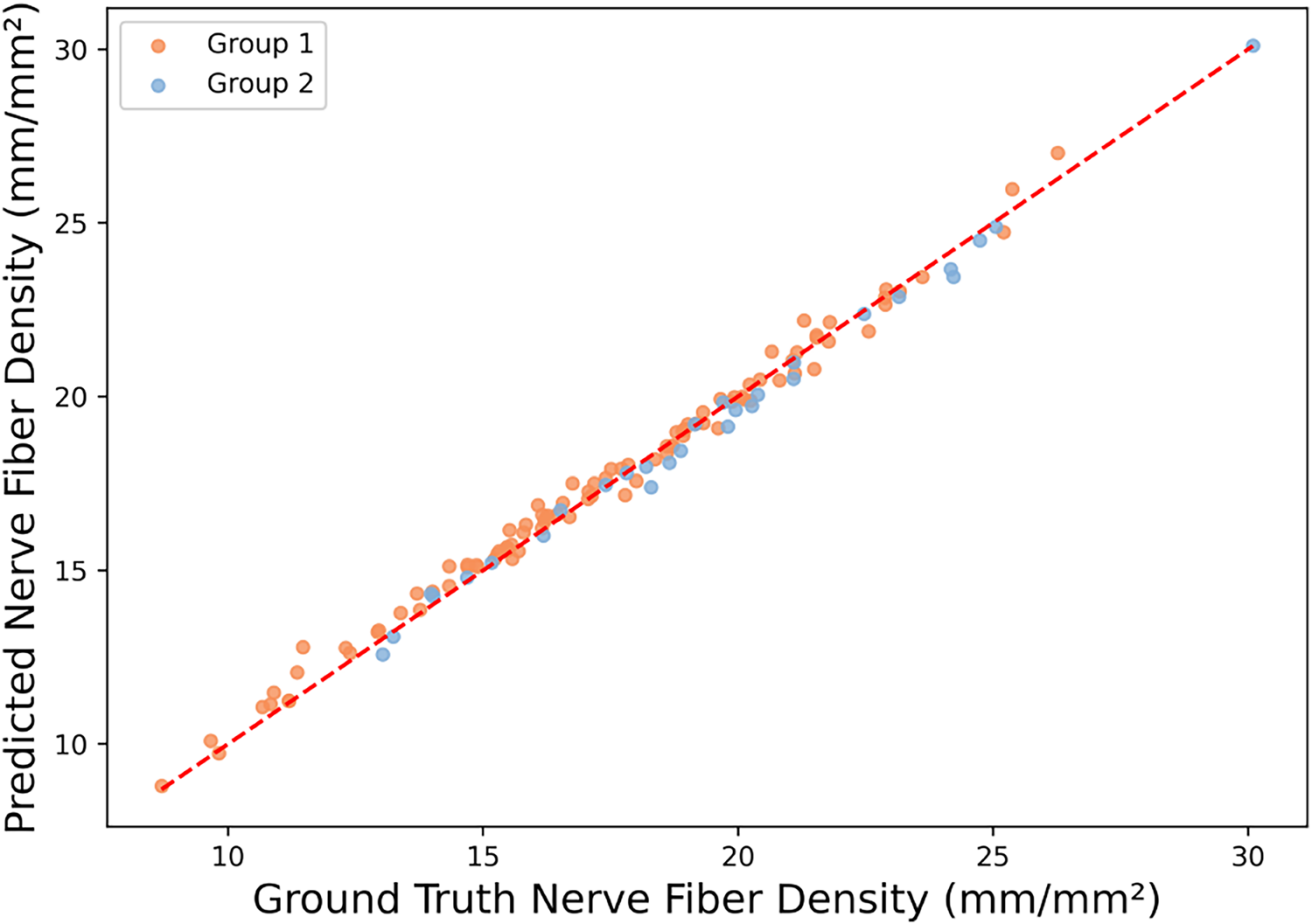
Manual method vs automated method in estimating per participant CNFL.

Figure 2 shows a comparison between the manual method (ground truth) and the automated method (CNFL automated pipeline) for estimating CNFL per each participant. Where each point on the figure represents the CNFL value for one participant. The close alignment of the data points along the red reference line indicates a strong agreement (1.79% MAPE and 0.30 MAE) between the automated estimates and the manually derived ground truth values.

Bland-Altman analyses were performed between the automated and manual methods to compare their agreement. On a per-image level, the automated CNFL estimation method had a bias of -0.01 mm, indicating a slight underestimation by the automated method. The standard deviation of the differences was 0.13 mm, resulting in 95% limits of agreement approximately between -0.26 and +0.23 mm. Points within the range are clustered tightly, and the outlier points do not have large errors.

On a per participant level, the automated CNFL estimation method had a bias of -0.07 $\hbox {mm/mm}^2$, indicating a slight underestimation compared to the manual method. The 95% limits of agreement ranged from -0.80 to +0.66 $\hbox {mm/mm}^2$. The differences were evenly distributed around the mean with no apparent trend, indicating that the error was consistent across the range of density values. A few outliers above the upper limit reflect occasional overestimation. Clinically, discrepancies of less than 1 $\hbox {mm/mm}^2$ are considered irrelevant, unless large cohorts with hundreds of subjects are considered, which is larger than IVCM studies typically comprise.

Plots of both analyses are available in Supplementary Materials.

#### DC density estimation

The metrics presented in Table 1 indicate acceptable performance for the DC segmentation model. However, the metrics are negatively exaggerated for positive predictions, since the positive classes (both with and without dendrites) only occupy small regions of the image. The pixel-wise metrics are therefore very sensitive to the small positive values, where a small, contextually insignificant change can lead to drastic changes in the metrics. This leads to low values that do not directly correlate to the counting performance of the model and algorithm. This exaggeration also leads to the extremely high specificity, because the errors in the negative class are overpowered by the large number of true negative predictions caused by the class imbalance.

**Fig. 3:**
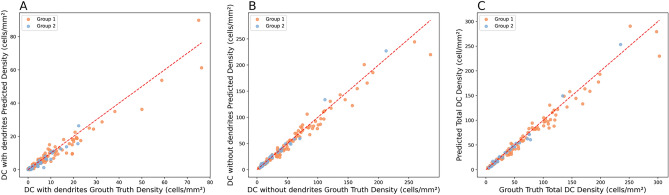
Automated method vs manual method in predicting per participant DC densities for the 2 cell types, and total density. Each point represents an individual participant. A - DC with dendrites. B - DC without dendrites. C - total DC density.

The two scatter plots shown in Fig. S18A and S18B in Supplementary Material illustrate the performance of the automated method in estimating counts of DC with dendrites and DC without dendrites. The DC segmentation model performed significantly better for DC without dendrites than for DC with dendrites, as evidenced by both the visual distribution of points and the error metrics. Specifically, the MAPE for DC with dendrites was 29.39% with an MAE of 0.44, while DC without dendrites had a considerably lower MAPE of 18.75% and MAE of 1.27. Although the MAE was numerically higher for DC without dendrites, as a result of the larger range of values, the relative error (MAPE) was substantially lower, indicating more accurate predictions in proportion to the available ground truth data. The tighter clustering of points around the diagonal line in the DC without dendrites plot (Fig. 3B) further supports the better consistency and reliability of the model for this type of DC.

Similar to the DC count results, the automated method performed better in predicting the density of DC without dendrites compared to DC with dendrites (see Fig. 3A and 3B). In particular, the prediction errors tended to increase with higher density values. This may be due to the difficulty of manually annotating every DC in images with very high DC density, which introduces uncertainty into the ground truth itself.

Shown in Fig. 3C, the performance of the total density is a mixture of DC with and without dendrites, because the relation of total density being a simple sum of densities of both types. The overall trend and distribution are similar for all three plots presented.

The Bland-Altman analyses, visually represented by their plots available in Supplementary Materials, demonstrates the agreement between the manual and automated methods for the two types of cells. For DC with dendrites, the mean difference was -1.07 $\hbox {cells/mm}^2$, indicating that the under/overestimation errors were distributed evenly. The 95% limits of agreement was -7.27 to 5.12 $\hbox {cells/mm}^2$, representing a difference of about ± 1 cell per image, which is acceptable. The distribution of the data points shows that the error for this type stays moderately consistent with some outliers, while at lower values, the predictions were accurate. For DC without dendrites, the mean difference was -2.31 $\hbox {cells/mm}^2$, showing that the bias was also low for this class. The 95% limits of agreement has a lower value of -20.94, and an upper value of 16.31 $\hbox {cells/mm}^2$, representing a difference of about ± 3 cells per image. The errors increased as the number of cells increased, this behavior was also observed in the previous analysis.

### Summary

Table 2 summarizes the performance of the automated pipelines for CNFL and DC density estimation, evaluated using MAPE and MAE. The results show that the automated CNFL pipeline achieved substantially lower errors (MAPE: 1.8%, MAE: 0.3 $\hbox {mm/mm}^2$) compared to the automated DC density pipeline (MAPE: 12.0%, MAE: 6.6 $\hbox {cells/mm}^2$), indicating higher accuracy and consistency in CNFL estimation. However, errors represent one cell or a few per image and are considered below the threshold of clinical significance. As one image is 0.16 $\hbox {mm}^2$, one cell per image would equate to 6.25 $\hbox {cells/mm}^2$. Thus, the errors on in DC estimation are clinically acceptable.

**Table 2:** Performance results for automated approaches.

	MAPE, %	MAE, CNFL: $\hbox {mm/mm}^2$, DC: $\hbox {cells/mm}^2$
Automated CNFL	1.8	0.3
Automated CNFL by ACCMetrics Tool	13.8	2.5
Automated DC (with dendrites) Density Pipeline	26.3	2.0
Automated DC (without dendrites) Density Pipeline	12.3	5.6
Automated Overall DC Density Pipeline	12.0	6.6

Table 3 summarizes the group-level statistics for CNFL as measured by the experienced observer performing annotations (manual method) and the automated method. The mean and standard deviation values are comparable across the sources. As given in the table, the difference in CNFL between Group 1 and Group 2 was statistically significant when measured by either method.

To assess the correlation between the manual and automated methods across participants, we computed the average intra-class correlation coefficient (ICC) between the model and experienced observer, which was 0.9951 with a 95% confidence interval of [0.9900, 1.0000], and the overall Pearson correlation coefficient (PCC) was 0.9958 with 95% CI of [0.9941, 0.9971].

Table 4 summarizes the overall and group-level statistics for DC density (with and without dendrites) as measured by the manual and the automated method. Using the manual method, the difference between the groups for DC with dendrites, without dendrites and total DC densities were statistically significant. The automated method produced similar findings.

**Table 3:** Group statistics for CNFL.

CNFL	Group 1 (ocular symptoms)	Group 2 (control)	Independent Samples t-Test* p*-value
	Mean ± SD (mm/mm^2^)	Mean ± SD (mm/mm^2^)	
Manual Method	17.3 ± 3.8	19.4 ± 4.0	0.012
Automated Method	17.5 ± 3.6	19.1 ± 4.0	0.034
ACCMetrics Tool	15.2 ± 3.3	16.0 ± 3.4	0.231

For the DC predictions, we calculated the ICCs and PCCs for each cell type, comparing the model with the experienced observer. For DC with dendrites, the ICC was 0.9631 95%CI: [0.9400, 0.9800], indicating high reliability. DC without dendrites produced slightly higher agreement, ICC was 0.9818 with 95% confidence intervals at [0.9700, 0.9900]. Pearson correlations are similarly strong, with 0.9676 and 95% CI of [0.9542, 0.9771] for DC with dendrites, 0.9839 and 95% CI of [0.9772, 0.9887] for DC without dendrites, respectively.

**Table 4:** Group statistics for DC density.

Dendritic Cells	Group 1		Group 2		Linear Mixed Model p-value		
	With	Without	With	Without	Density With Dendrites	Density Without Dendrites	Total Density
	Dendrites	Dendrites	Dendrites	Dendrites			
	Mean ± SD	Mean ± SD	Mean ± SD	Mean ± SD			
	($\hbox {cells/mm}^2$)	($\hbox {cells/mm}^2$)	($\hbox {cells/mm}^2$)	($\hbox {cells/mm}^2$)			
Manual Method	11.6 ± 14.1	59.8 ± 53.4	6.6 ± 6.0	36.0 ± 42.0	0.018	0.010	0.005
Automated Method	10.2 ± 13.1	56.2 ± 50.6	5.8 ± 6.1	37.3 ± 45.6	0.013	0.019	0.010

Effect size analyses showed that CNFL measurements demonstrated small-to-medium effects for both manual (d = 0.54) and automated (d = 0.45) approaches, indicating meaningful differences between Group 1 and Group 2. In contrast, all DC density measures showed very small effects (d < 0.2), suggesting minimal group differences. Overall, CNFL showed substantially greater discriminatory power than DC density, with the automated method closely matching manual performance.

## Discussion

This study compared a manual method for assessment of the corneal subbasal nerve plexus and dendritic cell density with an automated method combining deep learning segmentation and rule-based density estimation. The comparison was made in a sample of subjects experiencing persistent eye symptoms following mild COVID-19 infection, therefore, results represent a new solution for a real-world clinically relevant question. The current study is a step forward compared to previous studies that typically focused on segmentation accuracy rather than demonstrating clinical validity of their models through statistical comparisons between patient groups. In addition, in the current study we validated the new automatic method on a large dataset using group-based statistical analyses.

Our subbasal nerve segmentation model achieved a sensitivity of 0.82 and a specificity of 0.99 in detecting subbasal nerves relative to the ground truth manual tracing using NeuronJ in FIJI, which is comparable to previous studies^24–26^. The overall automated method for CNFL also demonstrated high agreement with the manual method, with a high ICC of 0.9951 and a high PCC of 0.9958. In comparison with the established automated CNFL method, ACCMetrics, our approach demonstrates improved accuracy (MAE:0.3). While ACCMetrics shows a tendency to underestimate nerve counts across both groups (Table 3) and yields a higher error (MAE: 2.5; Table 2), our method provides more precise estimations.

The DC model achieved a sensitivity of 0.50 and a specificity of 0.99. At first glance, this sensitivity appears lower than previously reported results^9^ (sensitivity 0.89, specificity 0.94). Hence, in our study, the segmentation model is used as an intermediate step toward DC density estimation, and we demonstrated that the cell-counting outcome remains robust despite lower pixel-level sensitivity. The difference between the mean DC densities from the manual method and the automated method for Group 1 was -1.1 $\hbox {cells/mm}^2$ for DC with dendrites, with 95% CI of [-1.78, -0.39]; -3.1 $\hbox {cells/mm}^2$ for DC without dendrites, with 95% CI of [-5.1, -1.0]. For Group 2, the difference was -1.0 $\hbox {cells/mm}^2$ for DC with dendrites, with 95% CI of [-1.79, -0.27]; 0.3 $\hbox {cells/mm}^2$ for DC without dendrites, with 95% CI of [-1.93, 2.60]. The differences here are small, as an error as high as 6.25 $\hbox {cells/mm}^2$ would correspond to one cell mistake per image.

Previous studies have employed various pre-processing techniques, including background removal and enhancing the contrast between nerve fibers and background^25^. Additionally, post-processing steps, such as morphological operations^9^, have been applied to reconstruct continuous nerve fiber lines and reduce noise caused by dendritic cells mistakenly identified as nerve fibers. Instead of relying on multiple rule-based pre- and post-processing algorithms, we implemented an additional model (LERA-Net) based on the encoder architecture of ResUNet^10^. The model was trained to directly estimate nerve fibers density from ground truth annotations. This approach compensates for segmentation errors, such as interrupted or fragmented nerve fiber lines, in a more robust and data-driven manner.

The automated method performed well for both DCs and CNFL, however, CNFL proved to be more challenging. Nerve fibers density estimation is highly sensitive to the accurate reconstruction of fiber length and continuity of the reconstruction. Any segmentation errors, such as breaks or interruptions in the nerve fiber lines, directly affect the density measurement. In contrast, DC density is based on simple object counting, regardless of their shape or size. Therefore, segmentation mistakes have a much smaller impact on DC density estimation, making the automated method more effective in this case.

The manual method revealed statistically significant differences in both DC and CNFL densities between Group 1 and Group 2. The automated method results were consistent with the manual method results and the differences between groups were also significant. Regarding CNFL results, the CNFL estimation pipeline produces segmentation errors as described above, but the errors are learned and offset by the LERA-Net model leading to high agreement with the experienced human observer and statistically significant differences between participants from Group 1 and Group 2.

### Validity limitations

One potential limitation is that the model was trained and evaluated using annotations from a single human observer. This may limit the generalizability of the findings to annotations from other observers. However, the goal of this study was to compare machine performance against a human expert, rather than to establish independence from human observers. In this context, using one expert was a deliberate methodological choice, since the study’s focus was on replicating human-machine agreement rather than human-human variability.

Another potential limitation is the relatively smaller control group (30 subjects) compared to the patient group (100 subjects), which may limit the ability to fully capture variability in the healthy population. However, this imbalance is less critical in the context of deep-learning-based segmentation, where sample size is determined at the image and object level rather than the subject level, and our dataset contains substantially more nerve and DC images than comparable studies^9,11,27,28^, providing sufficient object-level variability for robust model training and evaluation. Furthermore, we applied appropriate statistical analyses (independent t-tests and linear mixed models) to demonstrate that the automated approach yields statistically meaningful measurements; these analyses were not intended for hypothesis testing, which would require balanced subject groups. Finally, while the clinical dataset served as a relevant use case for evaluating human-machine agreement, further validation across additional graders and imaging devices will be important to support broader applicability.

Static IVCM imaging does not allow differentiation between individual migrating cells and structures that are closely apposed to nerves or other cells. Since dendritic cells can move during image acquisition, their appearance may occasionally overlap or blur with adjacent static structures, making interpretation challenging. This limitation of static IVCM has been highlighted in previous time-lapse studies^29^, which demonstrates the importance of considering cell dynamics when interpreting single-frame images. From the perspective of our study, this limitation affects both human observers and the automated system, as both rely on static IVCM frames. Future integration of time-lapse or complementary imaging could help resolve these ambiguities and support more definitive interpretation.

### Future research

A manual quantification of IVCM images has some challenges and limitations: selection of representative images for nerve fibers and dendritic cells across observers is variable and subjective, and the challenge of annotating dendritic cells in images with a high cell density. The proposed automated method still relies on the manual selection of representative images, which is time consuming (3-5 minutes per patient or longer) and introduces potential bias. By automating this process, it becomes possible to grade a larger number of representative images per eye, thereby reducing selection bias. Automation would allow the system to include all images that meet expert-defined criteria, such as high quality (well-focused, good contrast/illumination), non-overlapping regions, and the highest dendritic cells density areas, with equivalent selection rules applied for nerve fibers^30^.

## Conclusion

This study compares manual with an automated (deep-learning based) method for corneal nerve fiber and dendritic cell density estimation in a sample of subjects experiencing persistent eye symptoms following mild COVID-19 infection and a control group. The automated method showed excellent agreement with manual method measurements and significantly reduced the analysis time. Overall, the automated approach reduced analysis time to 0.72 s/image for CNFL and 0.05 s/image for DC, compared to approximately 120 s/image with the manual method, representing an over 100-fold improvement in speed. This would enable direct access to results in the clinic for real-time decision-making, which is not implemented in current clinical workflows due to time constraints. For CNFL estimation, the deep learning model reiterated the statistically significant groupwise results and strong agreement with manual annotations. However, its sensitivity to segmentation errors, particularly in nerve fiber length estimation, highlights the need for further validation on independent datasets to ensure generalizability. The DC model reliably estimated DC without dendrites, but performance for DC with dendrites was worse than DC without dendrites and CNFL, due to class imbalance and morphological variability, suggesting the need for additional training data. The proposed automated method has been integrated into the IVCMAssist system to enable rapid, objective, and scalable analysis of corneal nerves and dendritic cells in raw IVCM images. The IVCMAssist system can already support clinical studies and decision-making based on quantified parameters at the patient level.

## Supplementary Information

Supplementary Information.

## Data Availability

The datasets analyzed in this study cannot be publicly shared due to ethical considerations and privacy agreements stipulated by the approval of the Swedish Ethical Review Authority; however, they may be made available upon reasonable request by contacting N.L.
