# Supplementary material for: Deep learning-based segmentation and density estimation of corneal nerves and dendritic cells from In Vivo confocal microscopy images
Source: Sci Rep. 2026 Jan 13;16:1620. doi: 10.1038/s41598-025-34412-6 (PMC12800303; doi:10.1038/s41598-025-34412-6)
Supplement: Supplementary file 1 — Supplementary Information. [file 41598_2025_34412_MOESM1_ESM.docx]

## Supplementary Methodology

#### Segmentation Model Architecture (CNFL Model and DC Model)

**Encoder-Decoder Structure**

The networks follow a symmetric encoder-decoder structure with levels of resolution (CNFL: 4 levels, DC: 5 levels). The encoder pathway progressively reduces spatial dimensions while increasing feature depth, extracting increasingly abstract representations of the subbasal nerve fibers:

- *Initial level:* Processes input images (CNFL: 384×384×2, DC:384×384×3) through a residual convolutional block with 64 filters for CNFL, 32 filters for DC.
- *Intermediate levels:* Successive downsampling operations via max pooling, each followed by residual blocks with doubling filter counts (CNFL: 128, 256, 512; DC: 64, 128, 256, 512).
- *Bridge section:* A bottleneck that processes the most abstract features with 1024 filters.
- *Decoder pathway:* Symmetrical upsampling levels with decreasing numbers of filters that gradually restore spatial resolution while integrating contextual information (CNFL: 512, 256, 128, 64; DC: 512, 256, 128, 64, 32).

**Residual Convolutional Blocks**

The fundamental building block of the architecture is the residual convolutional block (RCB), which addresses the degradation problem in deep networks. Each block consists of:

- A main path with two sequential 3×3 convolutional layers, each followed by batch normalization
- A parallel shortcut connection implemented as a 1×1 convolutional projection to match dimensions
- An element-wise addition operation that combines the main and shortcut paths
- A final ReLU activation function

This design enables gradient flow directly through the network during backpropagation, facilitating the training of deeper networks while maintaining feature fidelity, which enhances the preservation of the corneal nerve fibers’ delicate structures.

**Skip Connections**

A critical aspect of the architecture is the implementation of long-range skip connections between corresponding encoder and decoder levels. These connections are realized through concatenation operations that merge feature maps from encoder blocks with their counterparts in the decoder pathway. These skip connections serve multiple purposes in subbasal nerve fiber segmentation: (a) preserve fine spatial details that would otherwise be lost during downsampling; (b) facilitate gradient flow during training; (c) enable precise localization of nerve fibers structures while maintaining contextual awareness, and (d) combines low-level features (edges, textures) with high-level abstract representations.

**Output Layer**

The final layer of the network utilizes a 1×1 convolutional operation with softmax activation to produce a probability map across two channels (background and nerve fibers class) for the CNFL model. This binary segmentation approach was selected to match the clinical task of distinguishing subbasal nerve fibers from surrounding corneal structures.

For the DC model, the output layer utilizes the same 1×1 convolutional operation with softmax activation, but it produces a three-channel probability map that corresponds to the background, and the 2 DC classes.

#### LERA-Net Architecture

The architecture of LERA-Net starts with an input processing block that is a modified RCB. Input processing accepts single-channel binary mask images (384×384×1 pixels) as shown in Figure S3, and processes through a large 7×7 convolution with stride 2, followed by batch normalization, ReLU activation, and max pooling. This is then followed by the main pathway containing four sequential RCBs for feature extraction:

- 1. Two RCBs with 64 filters + max pooling
  2. Two RCBs with 128 filters + max pooling
  3. Two RCBs with 256 filters + max pooling
  4. Two RCBs with 512 filters (no pooling)

Attention mechanisms with both channel and spatial attention are then used to learn the importance of the feature maps.^1^ Channel-wise relationships are modelled through a Squeeze-and-Excitation (SE) block, a specialized attention mechanism that operates on feature channels. The SE mechanism begins by aggregating spatial information via global average pooling, followed by a dimensionality-reducing transformation using a bottleneck structure with reduction ratio of 16. The excitation operation consists of two fully connected layers: the first with a ReLU activation function for dimensionality reduction, and the second with a sigmoid activation function that produces channel-specific weights. These weights are then applied to the original feature maps through channel-wise multiplication, dynamically recalibrating channel importance.

Spatial attention implements a complementary focus on spatial regions, with an 1×1 convolution with sigmoid activation generating a spatial attention map. Then an element-wise multiplication applies spatial weights to feature maps. Feature fusion uses parallel global average pooling and global max pooling to extract complementary feature representations. Both pooled features are concatenated to create a 1024-dimensional feature vector.

The regression head has three dense layers with decreasing dimensionality (512, 256, 128). Each dense layer is followed by batch normalization and dropout (rates: 0.4, 0.3, 0.2). The final dense layer has a single neuron with linear activation for regression output.

#### Training of CNFL and DC Segmentation Models

The CNFL model was trained using a dataset of paired IVCM images and expert-annotated subbasal nerve fiber masks (see [Figure S6](#km6hunquc11k)). The annotated nerve fibers for an image were processed from its corresponding *.ndf* file, producing a mask for the image with one-hot encoding. The masks are dual-channel 384*384 images, with pixel value 1 in the first channel representing the background, and value 1 in the second channel for representing the nerve fibers.

The training process used dice coefficient^2^ as the loss function with the following formula:

$DL=1-\Sigma_{i}(\frac{2\Sigma_{k}(y_{true,k,i}\times y_{pred,k,i})+\varepsilon}{\Sigma_{k}(y_{true,k,i})+\Sigma_{k}(y_{pred,k,i})+\varepsilon})$ (S1)

Where:

$DL$ is the dice loss

$y_{true,k,i}$ are the binary values (0 or 1) indicating whether pixel *k* truly belongs to class *i* in the ground truth mask

$y_{pred,k,i}$ is the predicted probability (between 0 and 1) that pixel *k* belongs to class *i*

$\Sigma_{k}$ is the summation over all pixels in an image

$\Sigma_{i}$ is the summation over both classes

$\varepsilon$ is a smoothing factor set to a value of 1.0 to prevent division by zero, and avoid numerical instability in this use case, where predictions and targets are sparse. If a smaller value, for example, 0.01, is used, small changes in the prediction could lead to gradient explosions during backpropagation.

An experiment was conducted using binary cross-entropy^3^ loss, which produced results that are less desirable than the presented dice coefficient-based loss.^2^ To prevent overfitting as the subbasal nerve fiber model approaches later epochs, the learning rate was reduced once validation loss during training only yielded negligible improvements, down to a minimum rate where the learning rate remained unchanged. An early stopping criterion was set to stop training once the model’s validation loss during training began to decline, with a 10-epoch grace period.

The dendritic cell model training incorporated similar techniques to enhance performance and generalization (see [Figure S7](#yrqod8k68mms)). The JSON files containing the polygon re-annotations were processed into masks for model training. Annotated polygons were drawn onto the masks with their respective class, represented through one-hot encoding. The masks are 3-channel 384*384 images, where one channel represents one class. Pixels with value 1 in the first channel represent the background, value 1 in the second channel represents the type 1 (mature) dendritic cells, and value 1 in the third channel represents the type 2 (immature) dendritic cells.

Early stopping and model checkpointing from the nerve subbasal fiber model were also applied in an identical manner to this model. Training of the dendritic cell model employs a weighted dice coefficient loss^4^ function with the following formula:

$WDL=1-\Sigma_{i}(w_{i}\times\frac{2\Sigma_{k}(y_{true,k,i}\times y_{pred,k,i})+\varepsilon}{\Sigma_{k}(y_{true,k,i})+\Sigma_{k}(y_{pred,k,i})+\varepsilon})$ (S2)

Where:

$WDL$ is the weighted dice loss

$y_{true,k,i}$ are the binary values (0 or 1) indicating whether pixel *k* truly belongs to class *i* in the ground truth mask

$y_{pred,k,i}$ is the predicted probability (between 0 and 1) that pixel *k* belongs to class *i*

$\Sigma_{k}$ is the summation over all pixels in an image

$\Sigma_{i}$ is the summation over all three classes

$w_{i}$ are the weights for all 3 classes

$\varepsilon$ is a smoothing factor set to a value of 1.0 with the same reasoning as the corresponding term in dice loss used in the nerves model.

The classes are weighted with $w_{i}$to address the imbalance due to the structures of interest occupying small fractions of the image area. The implementation assigned different weights to each class (background, type 1, and type 2 dendritic cells) to prioritize accurate segmentation of the cells.

Both models were trained with a batch size of 8 for up to 100 epochs, though early stopping typically triggered earlier based on validation loss plateauing at around 30 to 40 epochs. The models were then evaluated with 5-fold cross-validation on the whole dataset.^5^

#### LERA-Net Training

The model took segmentation output and ground truth lengths as input for training, as shown in [Figure S8](#sdru5q2cxljx).

The network used the Adam optimizer with an exponential learning rate decay schedule, starting at 0.001. For the loss function, rather than conventional mean squared error, the model used the Huber loss function defined in [formula (S3)](#7k068zh9el7g) to enhance robustness against potential outliers in the nerve length measurements.


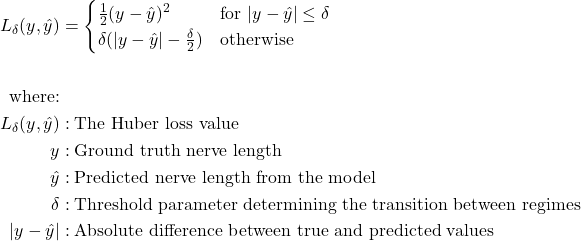
 (S3)

Multiple evaluation metrics were tracked during training, including loss, mean absolute percentage error (MAPE) and mean absolute error (MAE), to provide performance assessment. The training process incorporated two callback mechanisms to aim for optimal convergence: early stopping with a patience of 15 epochs to prevent overfitting, and model checkpointing to save the best-performing version based on validation loss. Additionally, all output values were normalized to a mean of 0 and standard deviation of 1 during training, the normalization parameters are preserved alongside the model for consistent inference on new data.

#### Automated Pipeline for CNFL and DC density

In the automated pipeline for patient-wise CNFL and DC estimation, selected images from each participant were first provided to a trained subbasal nerve fiber segmentation model as input. The models produced probability maps as the output, the maps are 2-channel for CNFL and 3-channel for DC. These maps are then post-processed using an argmax operation to generate binary masks, representing the segmented nerve structures or dendritic cells in the image. For CNFL, these binary nerve masks were subsequently input into the trained LERA-Net model, which estimated each image's nerve length in micrometers; and for DC, the cell counts are algorithmically obtained from the masks. Finally, the CNFL or DC density for the participant was calculated using [formula (1)](#jnbcgzv28tzo) or [formula (2)](#dvxgaeqa7m8w), [(3)](#dl6u4vubgaj6), [(4)](#9n9h5h6h24lb). The process of producing the patient-wise CNFL and DC metric from raw images is illustrated in [Figure S9](#7fb4s49frrbj).

- **Algorithmic Nerve Fiber Length Estimator in Python**. Estimating the CNFL from a prediction image needs to be done in a bespoke way since the model does not produce data used by the NeuronJ plugin to calculate lengths. The algorithmic length estimator calculates the total length of subbasal nerves in an image with the following steps:

1. The predicted nerve fibers in the binary prediction masks are reduced to one-pixel wide paths through morphological skeletonization.
2. Small branches and artifacts under a set length are removed by a pruning algorithm.
3. The pruned skeleton is traversed using connected component analysis, measuring each continuous nerve fiber segmented by counting pixels.
4. A scale factor (400/384 μm/pixel) is applied to convert pixel counts to micrometers. A factor of 1,000 is then used to express this value in mm.
5. Individual segment lengths are summed to calculate the total subbasal nerve length in the image.

- **Algorithmic Dendritic Cell Counter in Python**. The output masks of the dendritic cell model were saved in a format where predefined colors represent each class of cells. A counting algorithm processed the masks and gave an output of cell count for both classes. It follows these steps:

1. The segmented mask is loaded in grayscale format, containing pixel values of 0 (background), 128 (type 1 cells), and 255 (type 2 cells).
2. Two separate binary masks are created — one identifying type 1 cells (pixels with value 128) and another for type 2 cells (pixels with values between 255).
3. Unique integer labels are assigned to each connected region in both binary masks.
4. Type 2 cell regions smaller than 15 pixels and type 1 cell regions smaller than 35 pixels are discarded to eliminate noise and artifacts.
5. Centroid coordinates of regions meeting the size thresholds are stored in separate lists for each cell type.
6. The final count for each dendritic cell type is determined by counting the number of centroids in their respective lists.

- **Length Estimator Residual Attention Network (LERA-Net) - Machine Learning Model for Nerve Fibers Length Estimation.** LERA-Net represents a specialized neural network architecture designed for the regression task of estimating nerve length from binary mask images. The architecture is loosely based on the encoder structure of ResUNet, with modification to its input and custom output layers.^6^ The custom network incorporated several deep learning techniques to obtain results that proved to be better than a pure algorithmic approach. Detailed information about this model can be found in the supplementary methodology section.

###### **Visualization of CNFL model’s segmentation**

As the segmentation models’ predictions are rather complex, we present the following section to give examples of their behavior. In the accurate cases presented in [Figure S10](#dqb5iumle92v), the model performs according to expectations and produces a result that is close to the annotations.

[Figure S11](#a266rclqsc3i) shows 2 cases where the nerve fibers segmentation model underperformed. From these and other similar cases, the model yielded errors of two distinct types. The first source of error was the presence of objects similar to the nerve fibers, e.g., some dendritic cells being mistaken for nerve fibers. The second cause of error was faint nerve fibers, where a human annotator was able to identify the nerve fiber through domain knowledge, whereas the model failed to infer such information based on pixel inputs.

###### **Visualization of DC model’s segmentation**

In the examples shown in [Figure S12](#vyunm9qhk9gx), the model performed as expected, producing good masks that were counted algorithmically to yield DC counts.

However, the DC model sometimes misidentified non-DC objects as DC, or missed actual DC; examples are shown in [Figure S13](#1o9863r0rs07). Most false positive cases occurred due to the object’s shape similarity to a DC; and false negative cases were often caused by faint cells, blurry borders, or overlap with other objects. In rare cases, some objects or sections could be segmented as the wrong positive class (actual type 1 segmented as type 2, vice versa), which would produce errors that are more heavily penalized by the weighted dice loss, since the positive classes were weighted heavier.

#### IVCMAssist extension

Our automated approach builds upon the IVCMAssist system developed in our previous study^7^, which processes images from the corneal subbasal nerve plexus layer and allows users to select specific images for density calculation. This decision support system (DSS) enables users to upload a folder containing IVCM images of the subbasal nerve plexus layer by dragging and dropping it into the designated area, as illustrated in [Figure S14](#ml3mtf64nxc).

Once the images are uploaded, the user can select representative images for CNFL or DC density estimation using the checkboxes in the upper left corner of each image. The selected images will appear under the Representative Selected Images tab in the DSS, as shown in [Figure S15](#s15) A and B. By clicking the “Estimate NF Density” or “Estimate DC Density” buttons, the DSS executes the implemented automated pipeline and displays an overview of the predictions, including CNFL estimation and DC density estimation.

Multiple selections can be made from the same set of uploaded images, and multiple densities can be calculated by the IVCMAssist system; this is presented in the form of a prediction history. A user may select any history entry at any time to view the selected images and their statistics.

To view the segmented nerve fibers and dendritic cells, the user can click on the relevant image. A dialog will open, displaying three views as shown in [Figure S16](#s16). The leftmost view is the original image for reference, the center view shows the model’s raw predictions for DCs, and the rightmost view shows the model’s raw predictions for nerve fibers. Both raw prediction views also present their respective image-wise statistics in the bottom right corner.

#### Supplementary Figures

**
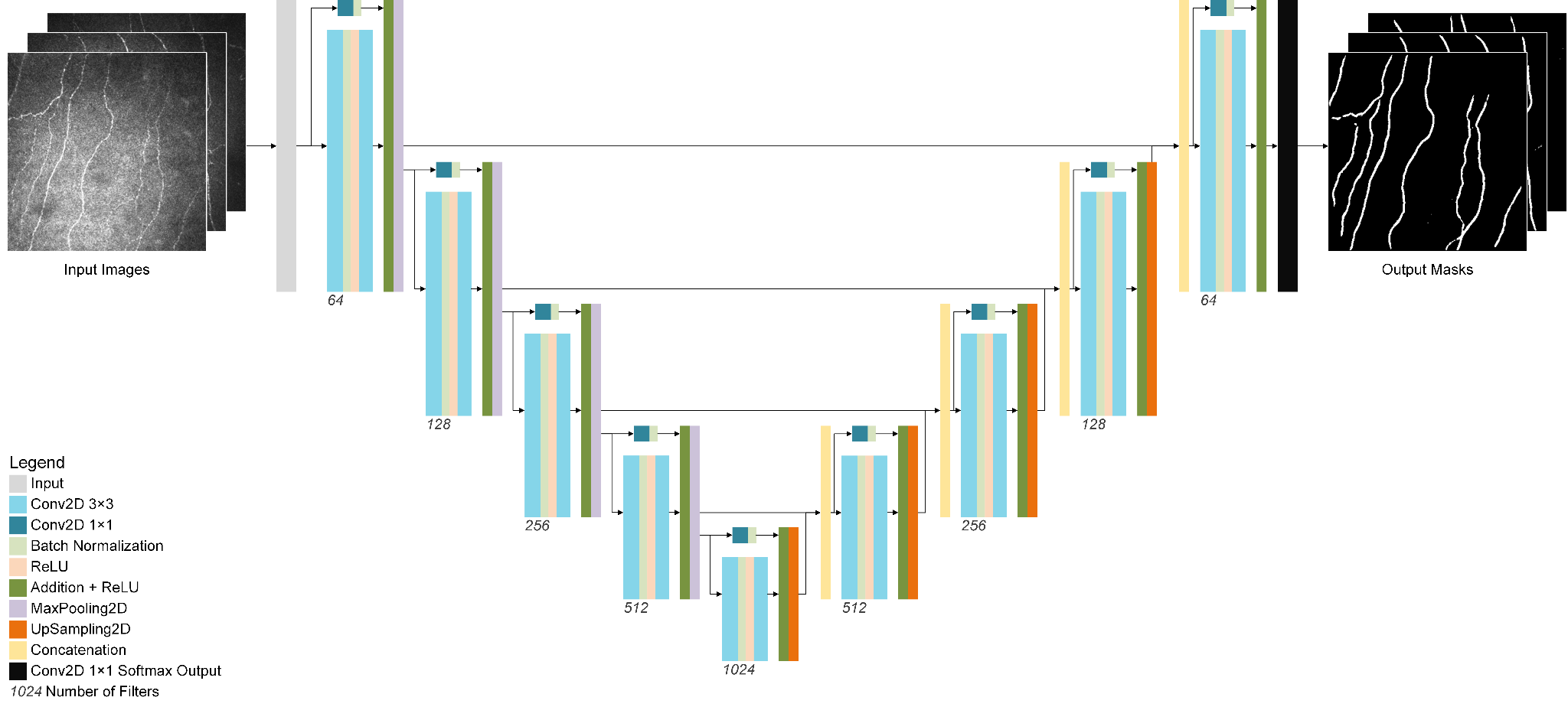
**

Figure S1. Corneal subbasal nerve fiber Segmentation Model Architecture


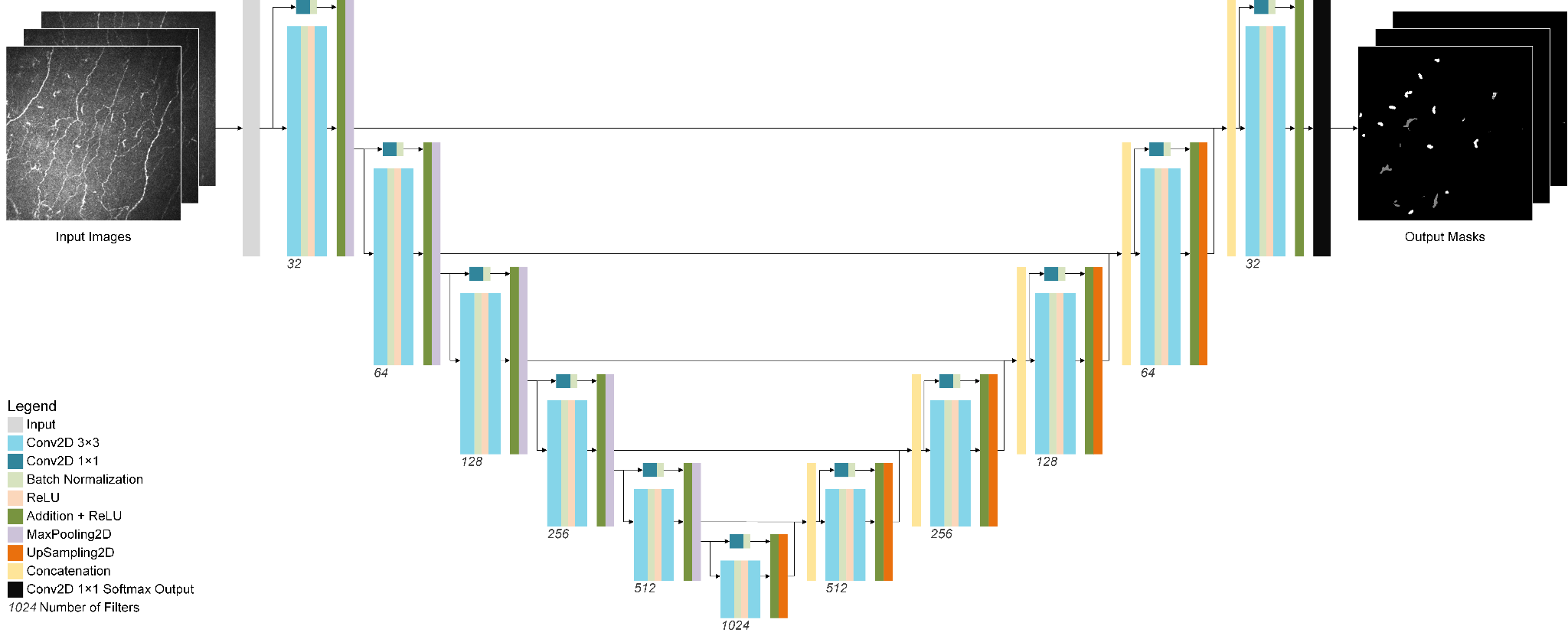


Figure S2. Dendritic Cell Segmentation Model Architecture


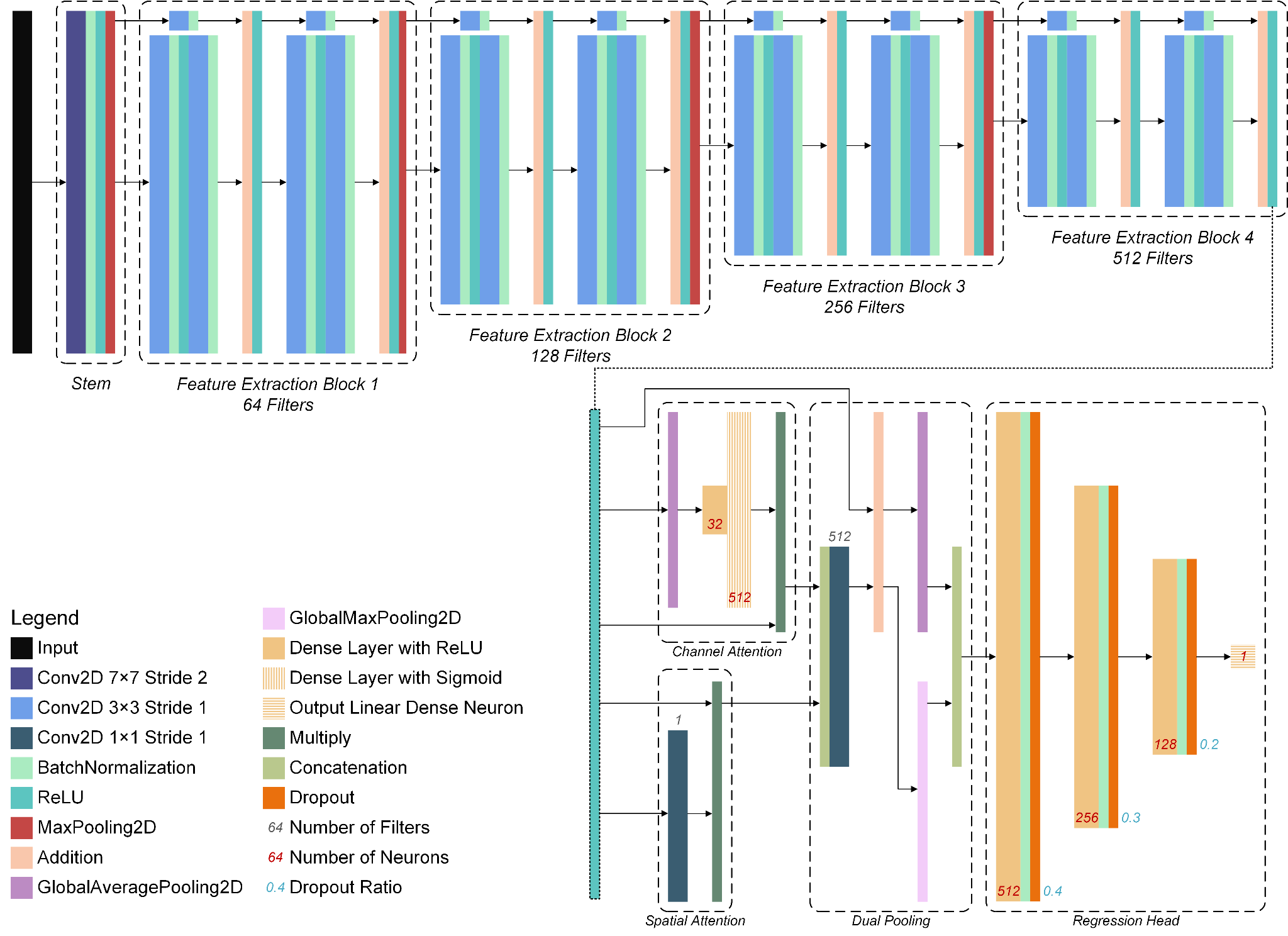


Figure S3. LERA-Net Architecture. The final ReLU layer of feature extraction block 4 is copied to the lower portion of the image to better show the attention, pooling and regression mechanisms.


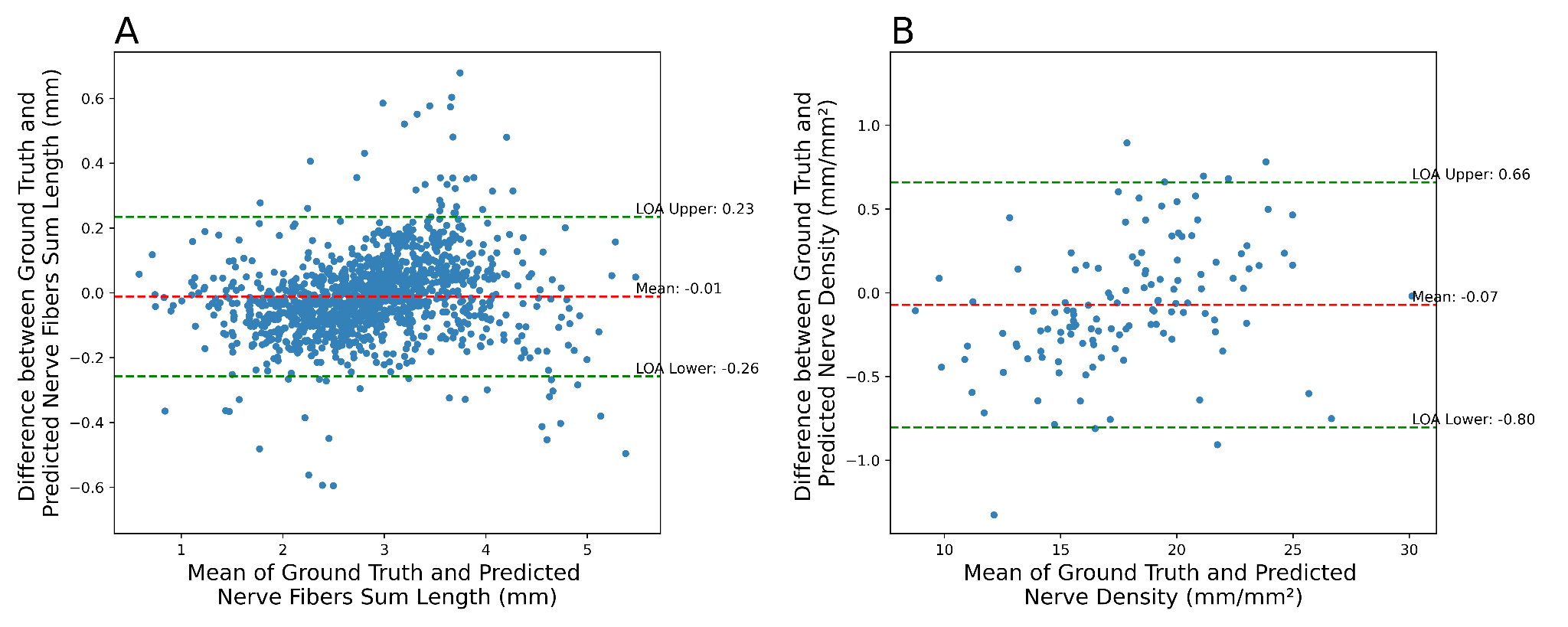


Figure S4. Bland-Altman plots evaluating the agreement between the automated method containing LERA-Net and the manual method for CNFL. A – per image results, B – per participant results.


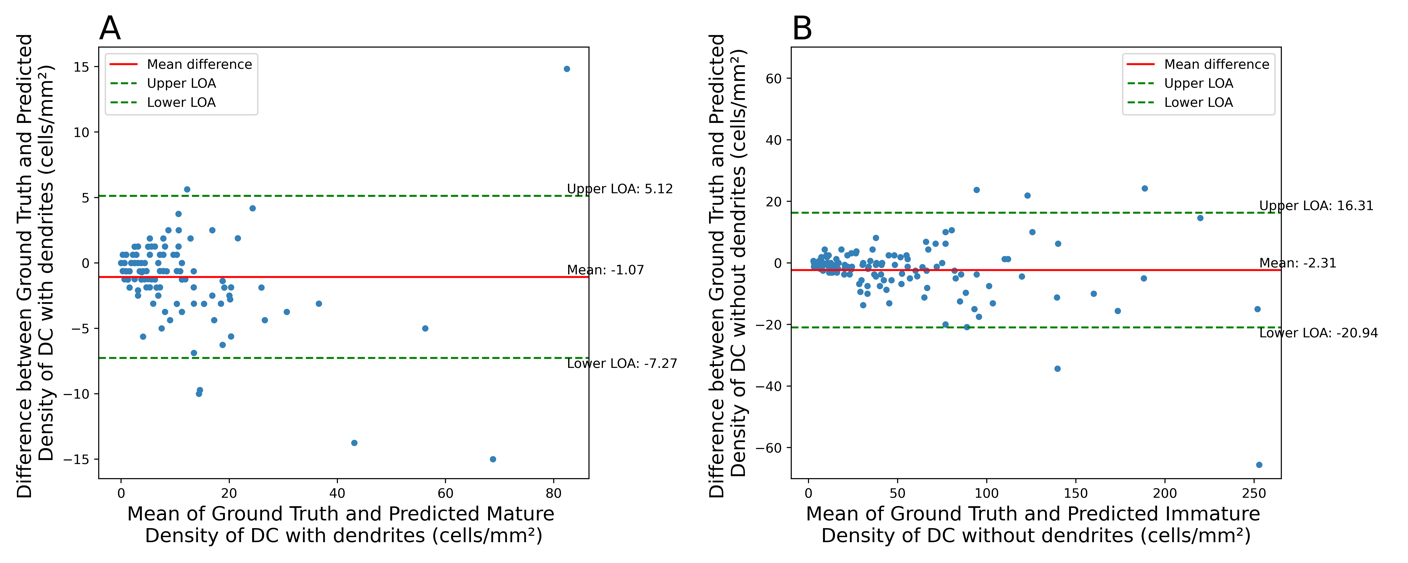


Figure S5. Bland-Altman plots evaluating the agreement between the automated method and the manual method for DC. A – DC with dendrites, B – DC without dendrites.


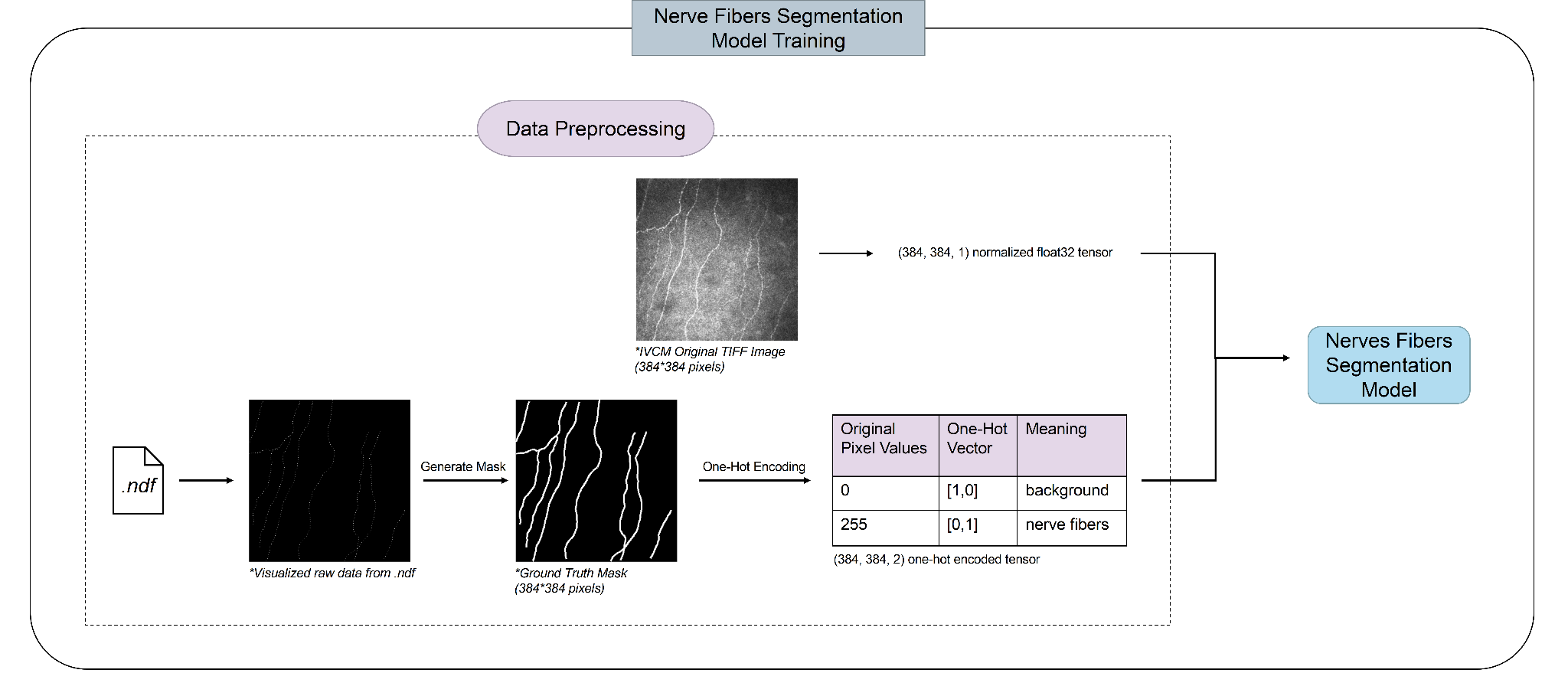


Figure S6. Subbasal Nerve Fibers Segmentation Model Training Pipeline. The data preprocessing steps prepare the data used to train the segmentation model.

**
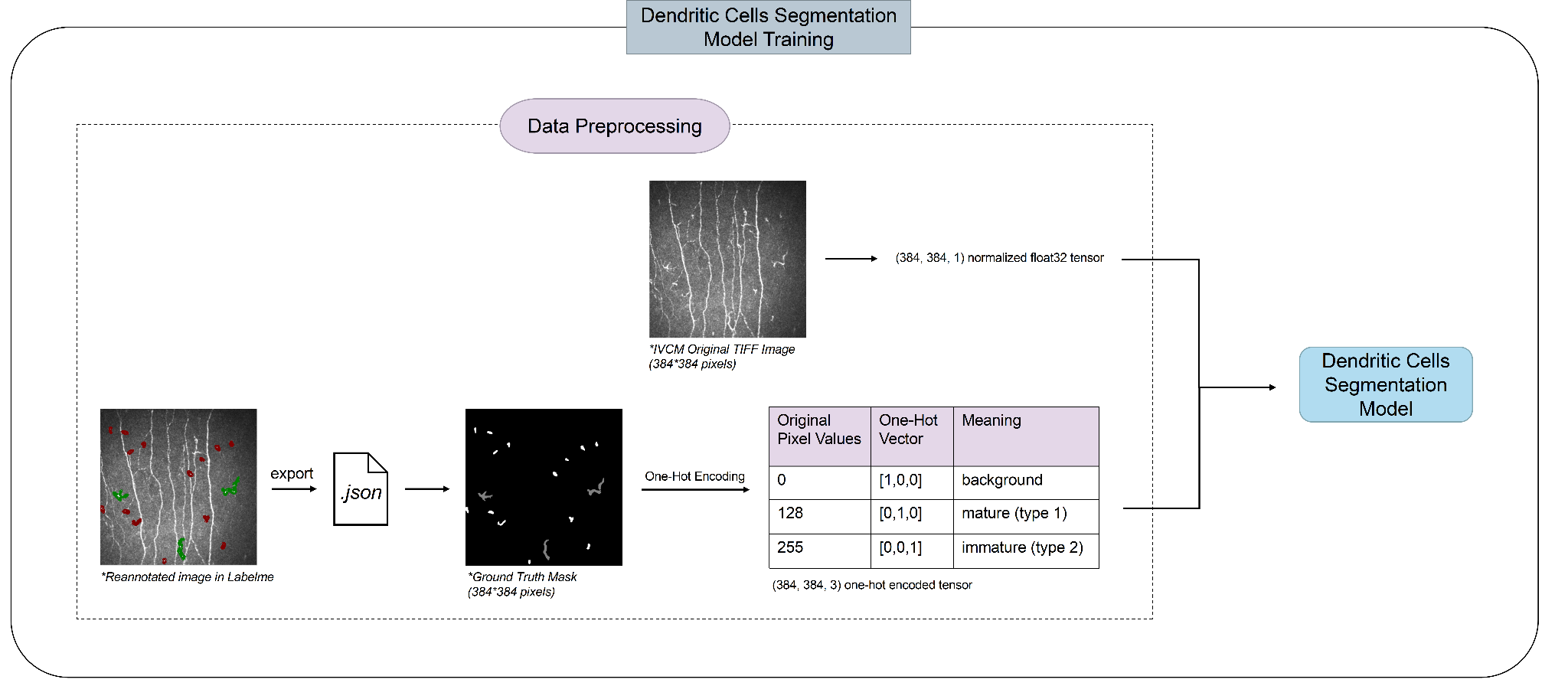
**

Figure S7. Dendritic Cell Segmentation Model Training Pipeline. The data preprocessing steps prepare the data used to train the segmentation model.


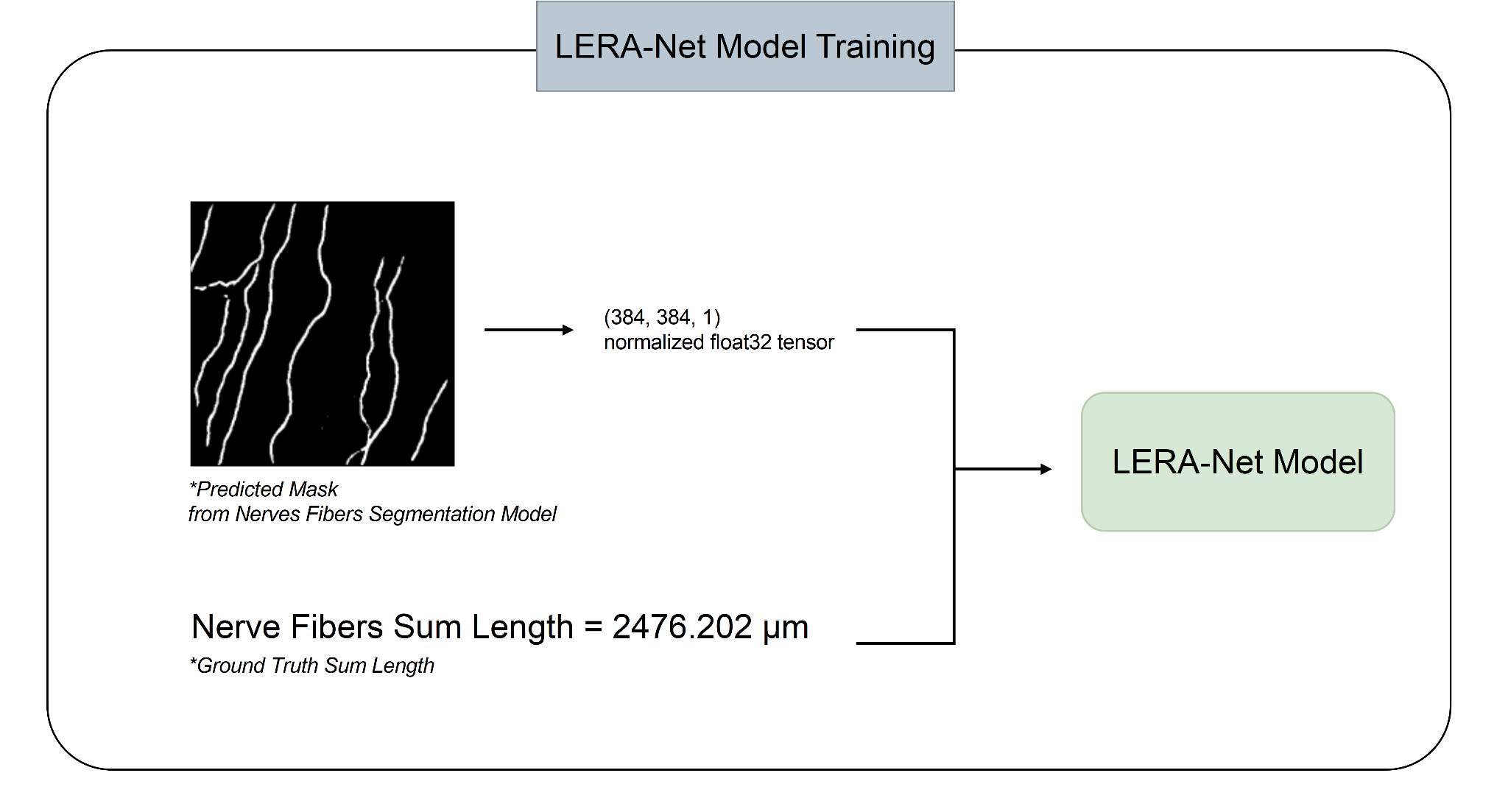


Figure S8. LERA-Net Model Training.


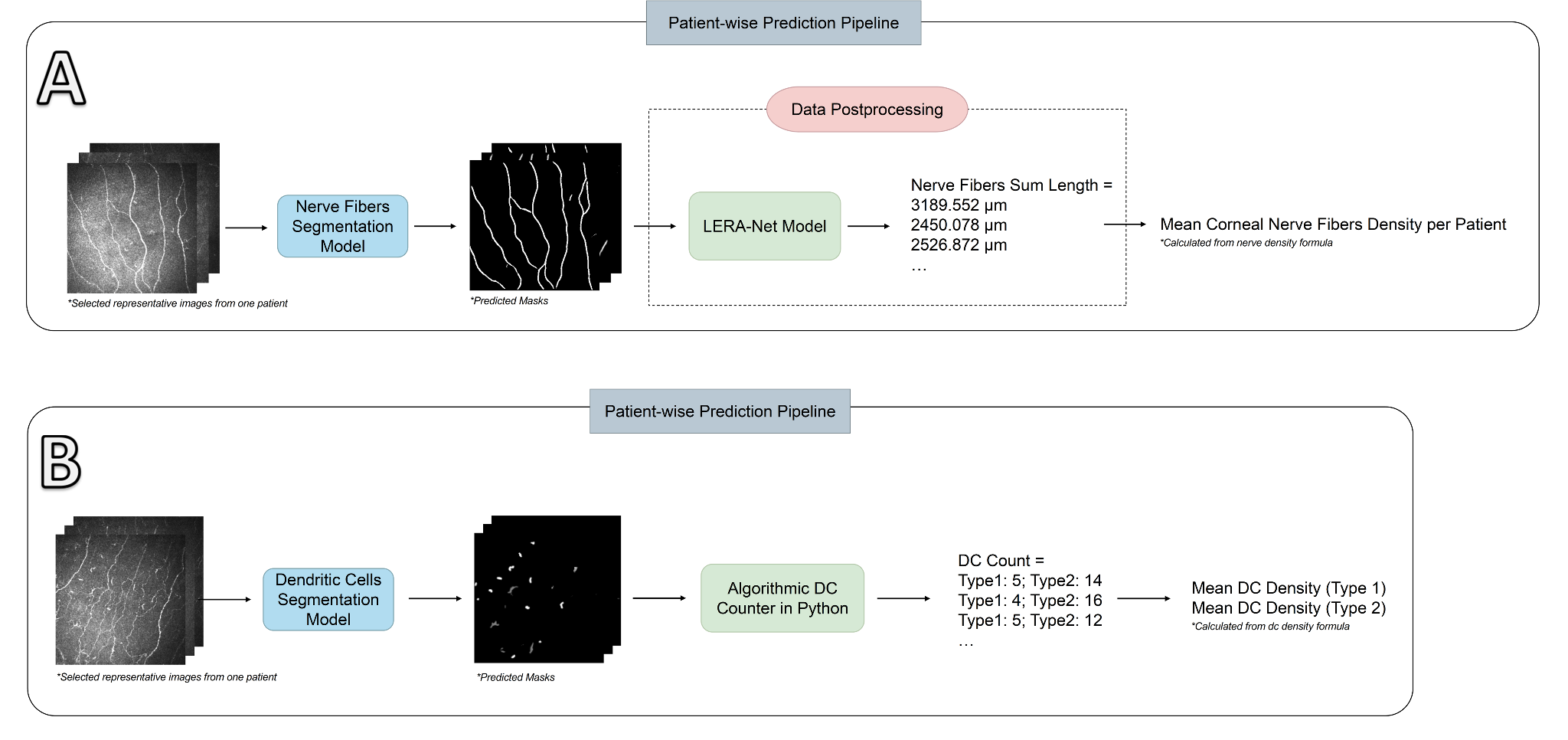


Figure S9. A – CNFL Estimation Pipeline. B – Dendritic Cell Density Estimation Pipeline.


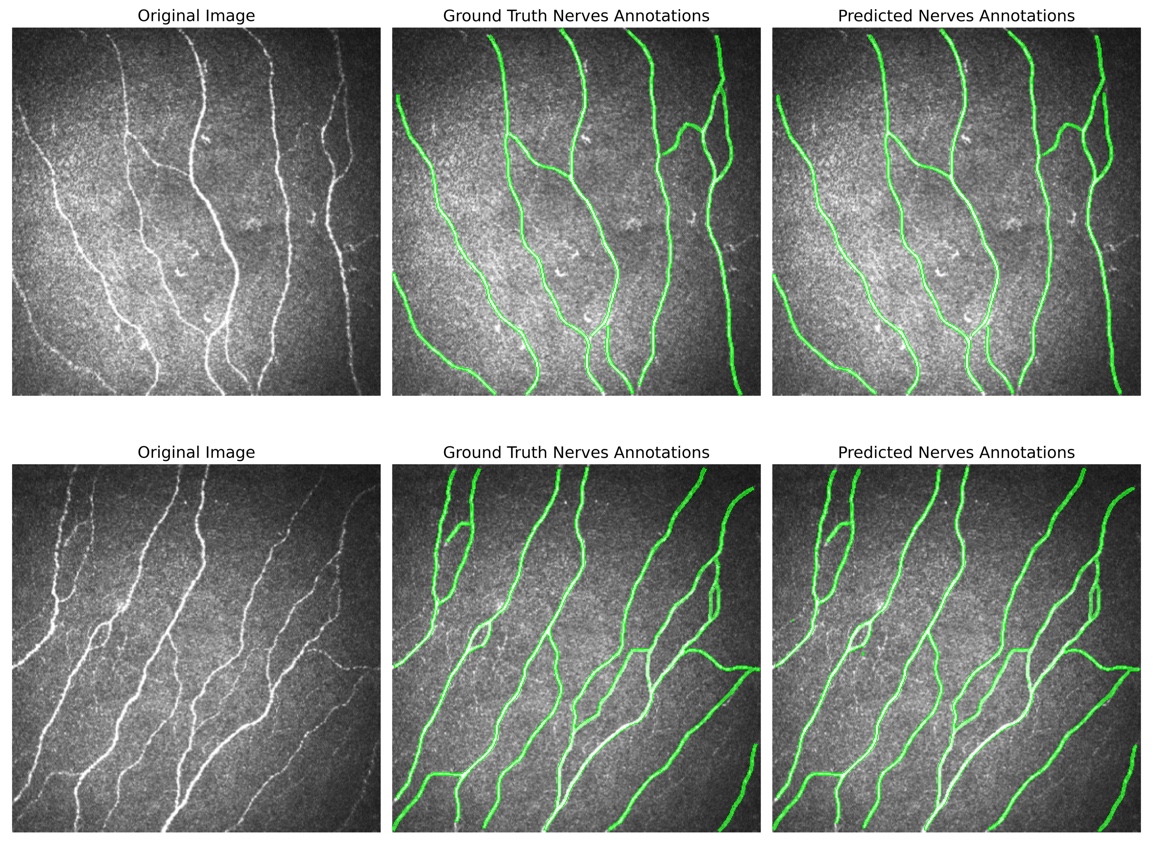


Figure S10. Accurate cases of nerve fibers segmentation model. Note that there still exist relatively faint nerves and objects that could be mistaken for nerve fibers, but the model shows its resilience in preventing these errors.


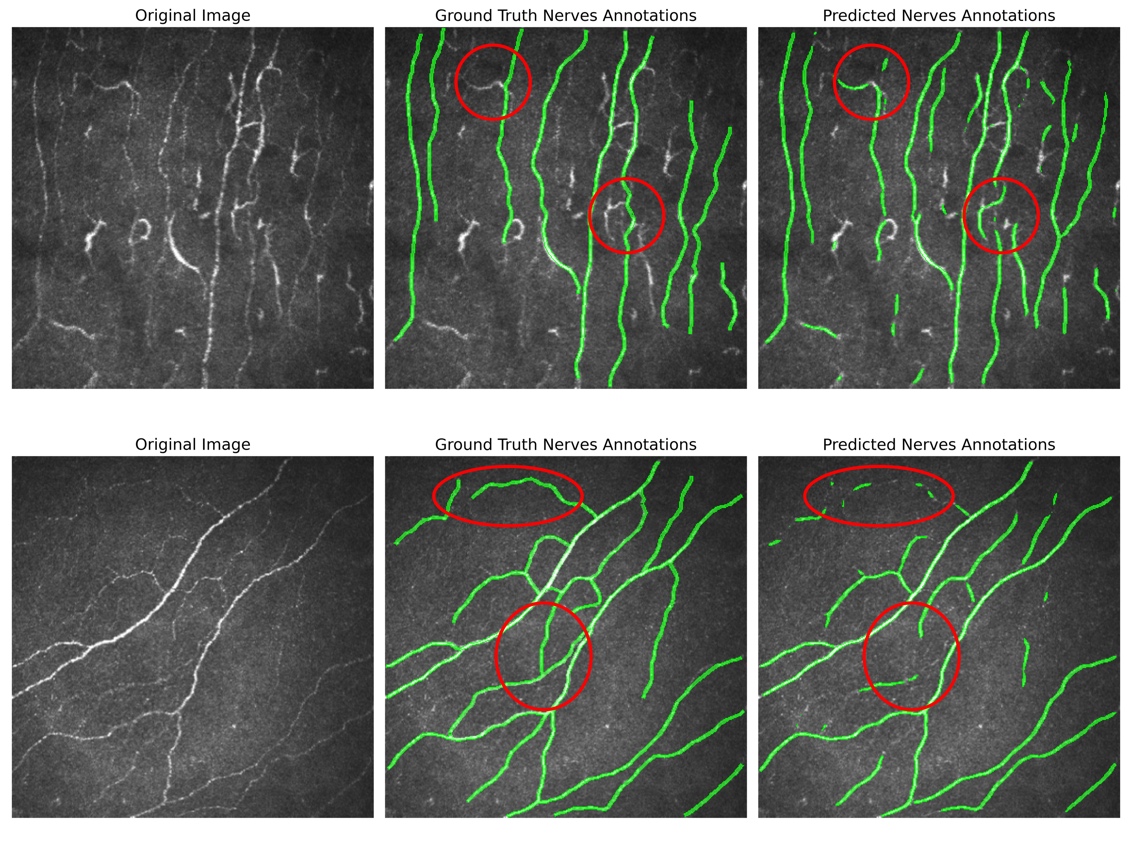


Figure S11. Inaccurate cases of nerve fibers segmentation model. The example above shows non-nerve fibers objects being falsely identified as nerve fibers, and the example below shows sections of faint nerve fibers not being identified.


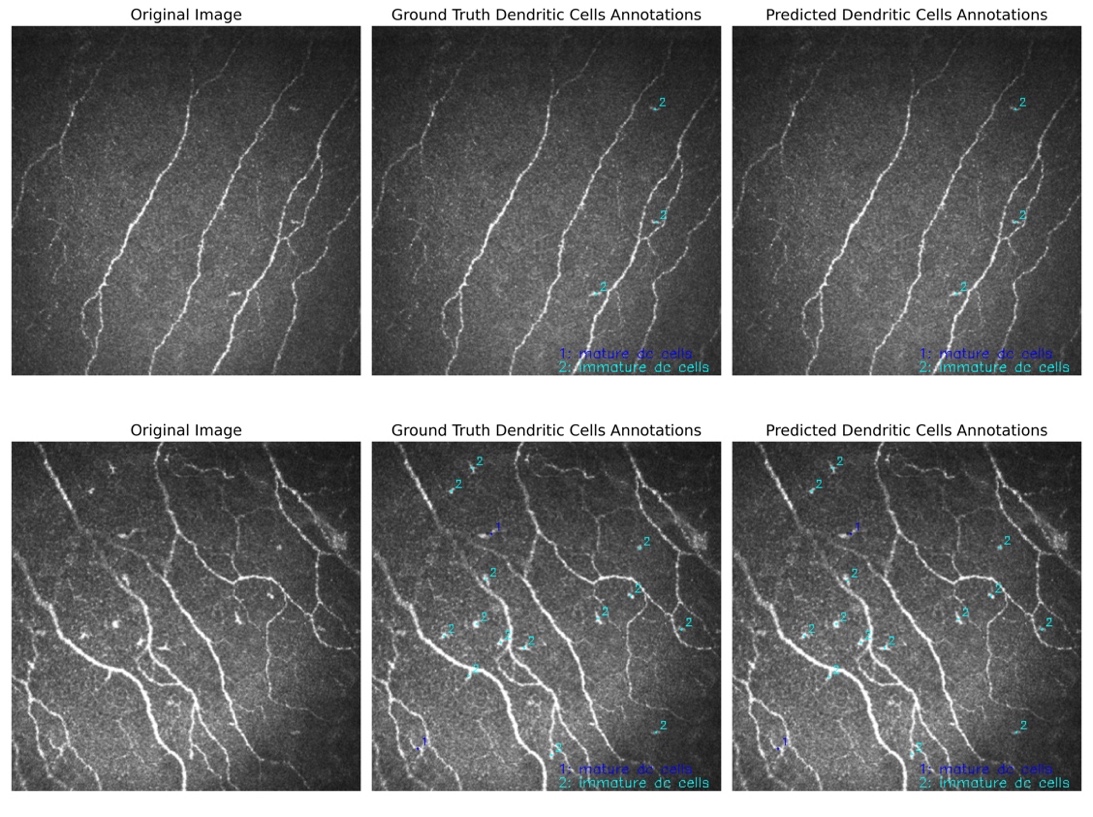


Figure S12. Accurate cases of DC segmentation model. The model performs well with different backgrounds and surrounding nerves layout.


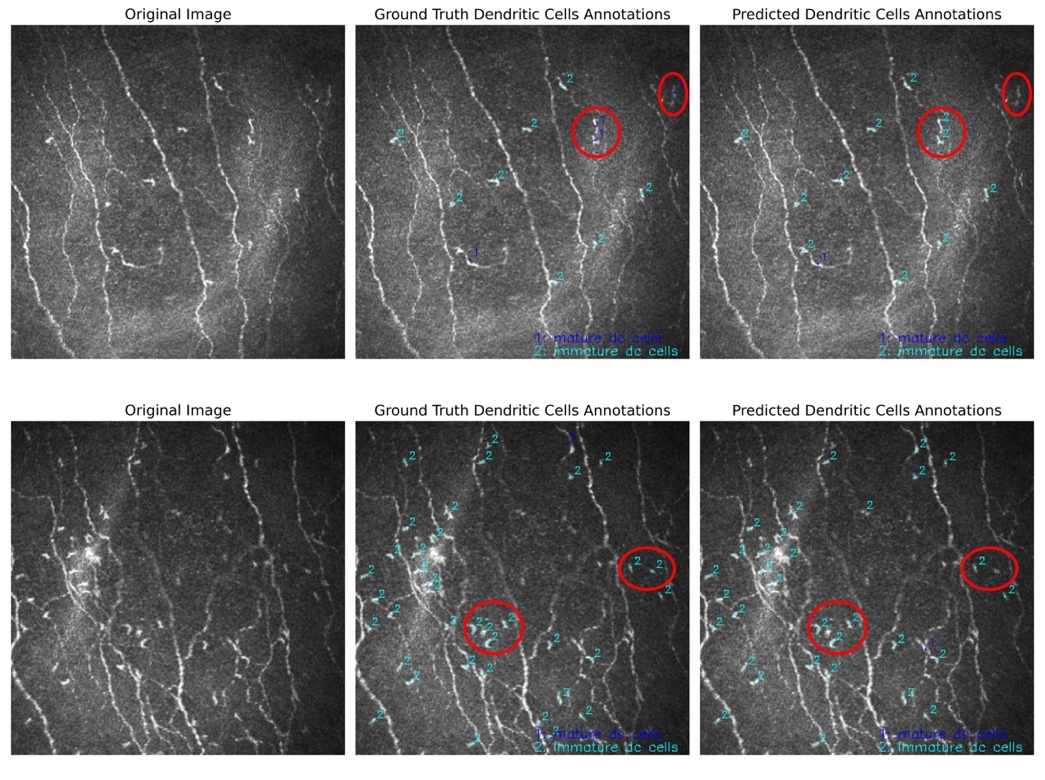


Figure S13. Inaccurate cases of the DC segmentation model. The example above shows some cells being identified as the wrong type while some are not classified at all. The example below shows cells not being classified properly.


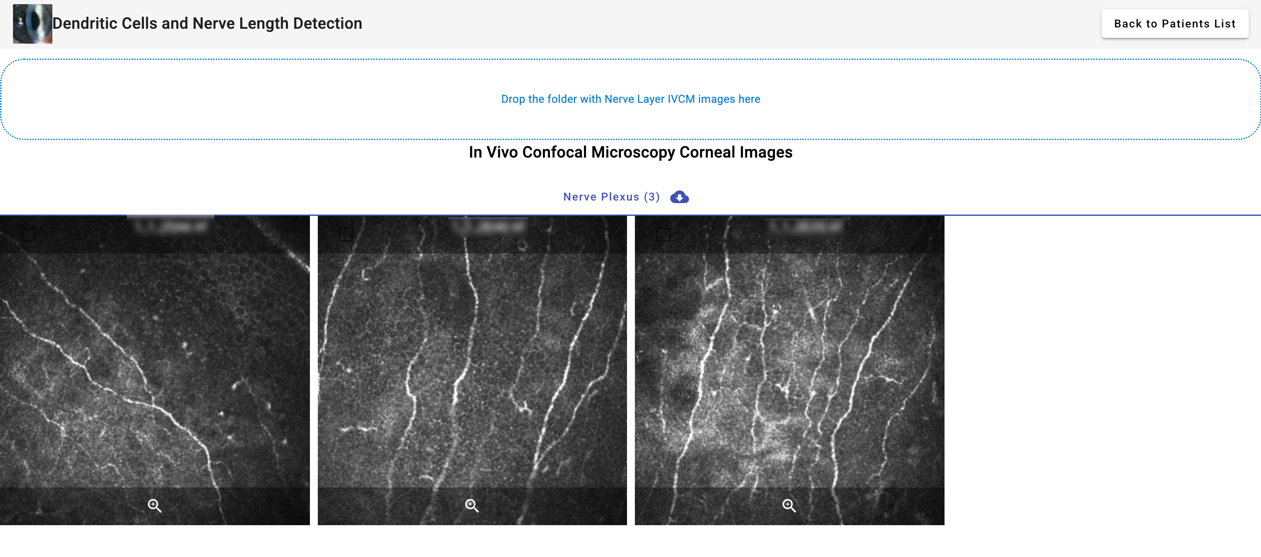


Figure S14. Uploading a folder of subbasal nerve plexus images into DSS.


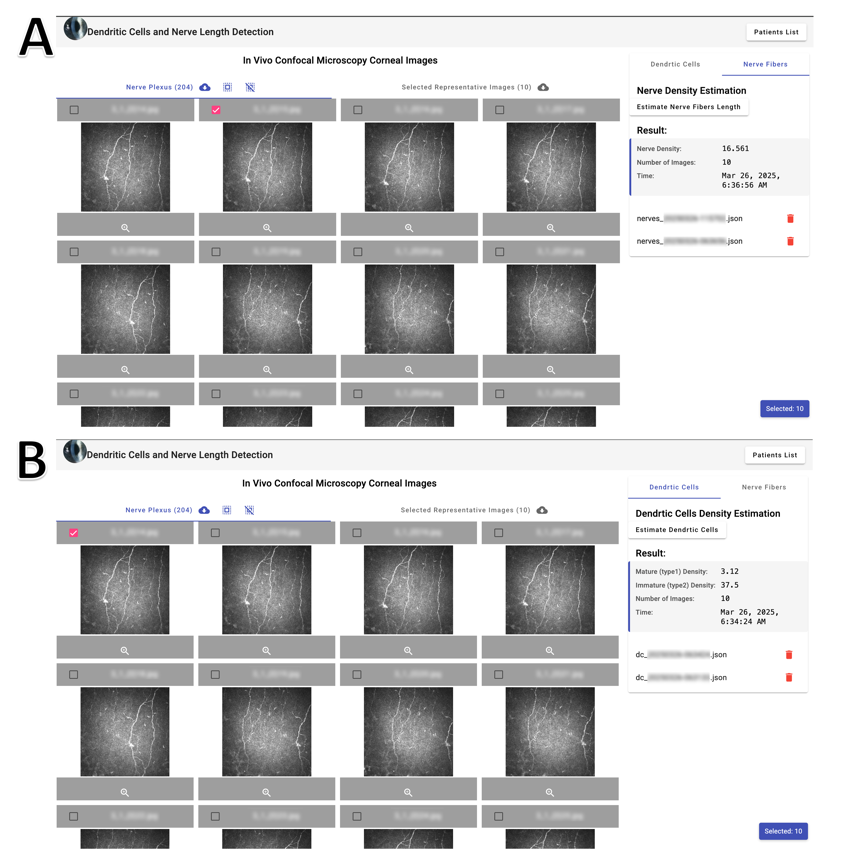


Figure S15. A - User Interface for CNFL estimation, B - User Interface for DC estimation.


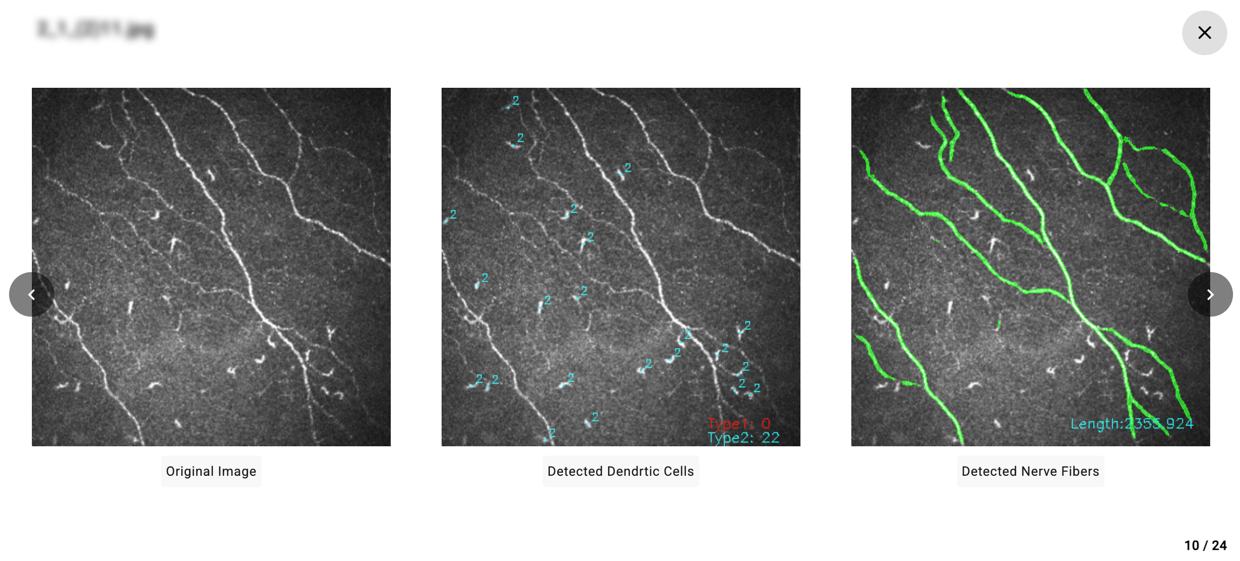


Figure S16. Displaying the nerve fibers and DC segmentation results in DSS.


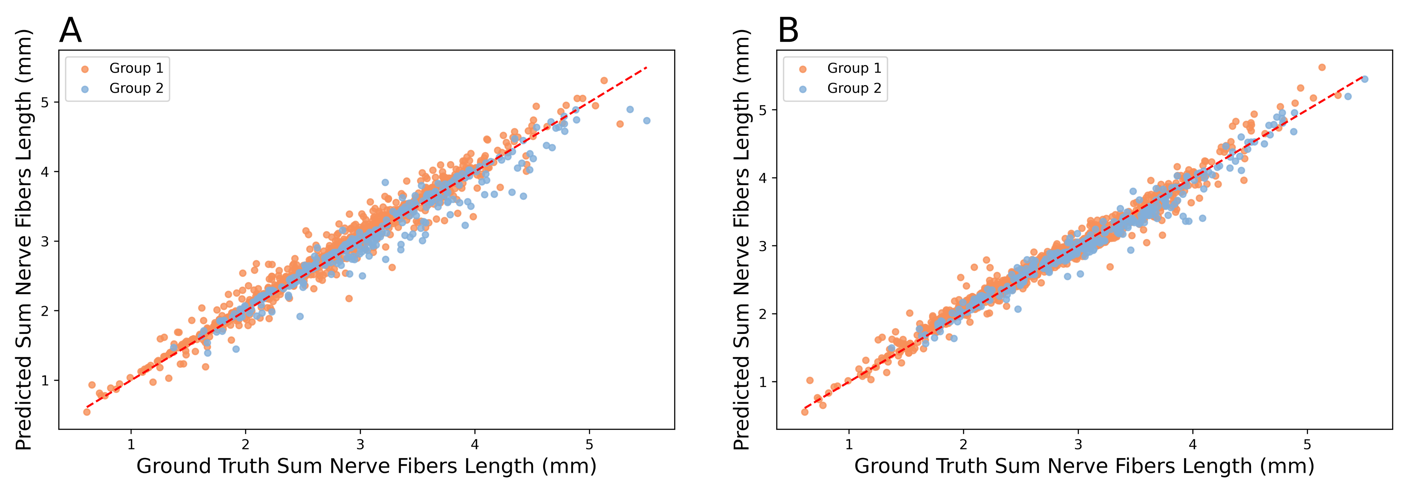


Figure S17. Comparison between the manual method to calculate CNFL per each image and the automated estimation methods. A – Manual vs Algorithmic approach, B – Manual vs Machine learning approach (LERA-Net).


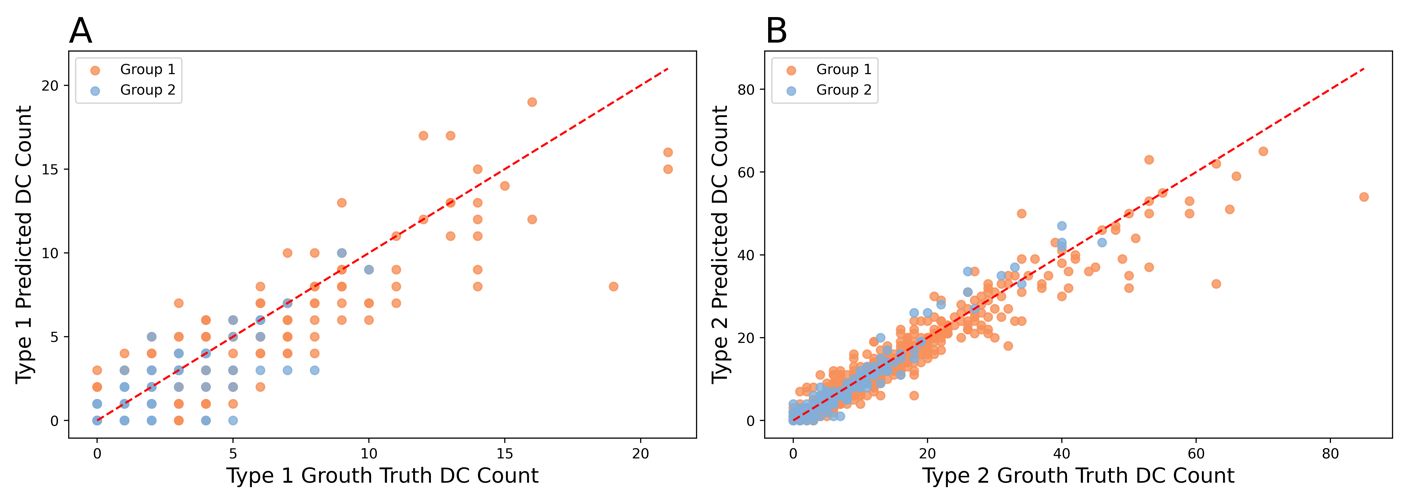


Figure S18. Automated method vs manual method in predicting per image DC counts for the 2 cell types, across two groups: Group 1 (blue) and Group 2 (orange). Each point represents an individual image, the red dashed line indicates the ideal prediction line (i.e., perfect agreement between predicted and actual counts). A – DC with dendrites. B – DC without dendrites

#### References

1 Oakley, J. D., Russakoff, D. B., McCarron, M. E., Weinberg, R. L., Izzi, J. M., Misra, S. L., ... & Mankowski, J. L. (2020). Deep learning-based analysis of macaque corneal sub-basal nerve fibers in confocal microscopy images. *Eye and Vision*, *7*, 1-11.

2 Mou, L., Zhao, Y., Chen, L., Cheng, J., Gu, Z., Hao, H., ... & Liu, J. (2019). CS-Net: Channel and spatial attention network for curvilinear structure segmentation. In Medical Image Computing and Computer Assisted Intervention–MICCAI 2019: 22nd International Conference, Shenzhen, China, October 13–17, 2019, Proceedings, Part I 22 (pp. 721-730). Springer International Publishing.

3 Schaldemose, E. L., Hammer, R. E., Ferdousi, M., Malik, R. A., Nyengaard, J. R., & Karlsson, P. (2020). An unbiased stereological method for corneal confocal microscopy in patients with diabetic polyneuropathy. *Scientific reports*, *10*(1), 12550.

4 Che, N. N., Jiang, Q. H., Ding, G. X., Chen, S. Y., Zhao, Z. X., Li, X., ... & Yang, H. Q. (2021). Corneal nerve fiber loss relates to cognitive impairment in patients with Parkinson’s disease. *npj Parkinson's Disease*, *7*(1), 80.

5 Qiao, Q., Xue, W., Li, J., Zheng, W., Yuan, Y., Li, C., ... & Hou, X. (2025). Automated program using convolutional neural networks for objective and reproducible selection of corneal confocal microscopy images. *Digital Health*, *11*, 20552076251326223.

6 Jha, D., Smedsrud, P. H., Riegler, M. A., Johansen, D., De Lange, T., Halvorsen, P., & Johansen, H. D. (2019, December). Resunet++: An advanced architecture for medical image segmentation. In *2019 IEEE international symposium on multimedia (ISM)* (pp. 225-2255). IEEE.

7 Lincke, A., Roth, J., Macedo, A. F., Bergman, P., Löwe, W., & Lagali, N. S. (2023). AI-Based decision-support system for diagnosing Acanthamoeba Keratitis using in vivo Confocal Microscopy images. *Translational vision science & technology*, *12*(11), 29-29.
